# Microbial Species-Area Relationships on the Skins of Amphibian Hosts

**DOI:** 10.1128/spectrum.01771-22

**Published:** 2023-03-30

**Authors:** Fan Yang, Zhidong Liu, Jin Zhou, Xuecheng Guo, Youhua Chen

**Affiliations:** a Chengdu Institute of Biology, Chinese Academy of Sciences, Chengdu, People’s Republic of China; b University of Chinese Academy of Science, Beijing, People’s Republic of China; Iowa State University

**Keywords:** generalized diversity measure, microbial diversity and distribution, ecological laws, change point analysis

## Abstract

Unlike species-area relationships (SARs) that have been widely reported for plants and animals on Earth, there is no clear understanding of the SARs for microorganisms. In this study, 358 specimens of 10 amphibian host species collected from the rural Chengdu region of southwest China were selected as island models for evaluating SAR curve shapes and assessing the skin microbiota from different amphibian species. The results showed that skin microbial diversity, measured using Hill’s number, presented significant differences between hosts, but the difference was insignificant between habitat-specific classifications of hosts. As for microbial SARs, other than the classical power-law (PL) model describing an expected steady increase in microbial diversity as sampled skin area increases, two additional trends were observed: (i) microbial diversity first rises and gradually decreases after reaching a maximum accrual diversity (MaxAD) and (ii) microbial diversity decreases and starts to rise after reaching the minimum accrual diversity (MinAD). Among the four SAR statistical models compared, it was consistently found that the models that can describe MaxAD were favorably selected in the highest frequency. Models that can describe MinAD and PL model also performed reasonably well. However, PL had the poorest fitting power, implying the necessity of introducing biologically meaningful complex SAR models in microbial diversity research. In conclusion, through multihost analyses, our study provided compelling evidence that microbial SARs are complex and nonlinear. A variety of ecological mechanisms may be used for explaining these, including, but not limited to, community saturation, small-island effects, or sampling heterogeneity.

**IMPORTANCE** In this study, we investigate species-area relationships (SARs) for skin-borne symbiotic microbes of wildlife hosts. Unlike the traditional SARs for plants and animals, symbiotic microbial SARs were complex. We found that both U-shaped and inverted U-shaped SAR models were widely favored for microbial taxa than the well-known power-law model in different host species. These favored models presented interesting statistical features, including minimal or maximal accrual diversity or inflection point. We provide intuitive derivations of these statistical properties. We showed that different habitat-specific amphibian hosts did not present distinct microbial diversity and skin-related SAR patterns. We predicted that approximately 600 to 1,400 cm^2^ (in two-dimensional [2D] measurement) or approximately 1,200 to 3 500 cm^2^ (in 3D measurement) are the skin area threshold range that can allow the emergence of minimal or maximal accrual microbial diversity with high chances. Finally, we list a variety of ecological mechanisms that may be used for explaining the observed nonlinear SAR trends.

## INTRODUCTION

The species-area relationship (SAR), i.e., the general increase in the number of species recorded with increasing sampling area, is one of the most widespread and investigated patterns in ecology (1). This relationship has facilitated the development of major theories in ecology, such as island biogeography theory (2) and many important developments in the fields of metapopulation biology, evolutionary ecology, and macroecology. In addition, conservation biologists frequently rely on the SAR to estimate global or regional biodiversity loss due to habitat loss and to develop strategies for conserving biological diversity (3–7).

Initially, the SAR was typically used for animal or plant macroecological studies. However, due to emergence of advanced genomic and, especially, metagenomic sequencing technologies, microbial ecologists are able to test major ecological theories of microbial ecology (8). The use of habitats with island-like attributes for studying microbial ecology has generated interest both for their potential as model systems and for understanding how the characteristics of microbes and their various community structures may affect their biogeographic patterns (9, 10). Despite their significant role in energy flow and material exchange within ecosystems, the microbial SAR is largely unknown. In microbial biogeography, it is urgent to clarify the usefulness of microbial SAR models and the underpinning mechanisms for understanding interactions between microbiomes and their host organisms.

According to island biogeography theory (2), larger islands tend to be richer in species because they have sufficient resources to support larger and more diverse populations; in this case, the large size makes every population less vulnerable to stochastic extinction. In contrast, if a small island were to sustain the same number of species as a larger island, the population size of each species must be smaller and therefore would be more vulnerable to stochastic extinctions (11). Many reports have suggested that the slopes and intercepts of the SAR curves depend significantly on the mean body size of the species. The number of small species typically show relatively higher local diversity, which increases more slowly with area than the number of larger species (12); this implies that microbial SARs may differ from conventional SARs for macroorganisms. However, questions arise regarding this topic. First, does microbial diversity follow the same basic rules as plant and animal biodiversity patterns, or does it follow different rules, limiting species according to different functional constraints? Many studies have found characteristic microbial SAR curves (13). Unfortunately, due to sampling effects and confusion of SAR with distance attenuation, the current microbial SAR research has not yet reached a consensus (14, 15). Second, the typical evenness in the size of populations for species of macroorganisms can also affect the shape and fit of the SAR (9), while the contributions of community evenness to the microbial SAR have yet to be explored across broad taxonomic groups within similar environments.

To solve these problems and advance the understanding of the microbial SAR, we need a framework that not only can eliminate the impact of sampling effects and the confusion between SAR and distance attenuation but also can overcome the serious limitation of the traditional SAR model, which is exclusively concerned about species richness while ignoring species abundance information. Islands are natural laboratories for SAR research and excellent research subjects for avoiding the effects of distance attenuation associated with studies in overlapping areas. For microorganisms, sampling effects cannot be avoided completely due to the size of the “island,” or the amount of effort and costs involved in sampling. Therefore, simulated island systems that can monitor microbial biodiversity exhaustively should be used for research. Ma (16) extended the traditional SAR models to diversity-area relationship (DAR) models by using Hill numbers as a general diversity metric system. With the extension, SAR is no longer limited to scaling relationships between species richness (number of species) and area and can consider both species richness and abundance to incorporate a broader perspective on community diversity. Hill numbers could be used as generalized diversity indices for microbial research.

In this study, amphibian hosts were selected as island models for studying the complex microbial DARs. Animal hosts were chosen for this study because an animal host skin is semi-isolated, dynamic, and can interact with surrounding abiotic environments. They are ideal as island models with finite size (skin surface area). Furthermore, amphibians are small-body-size animals, allowing us to sample the entire “island” to comprehensively document the skin microbiota without sampling artifacts. We hypothesized that microbial taxa on the skin of an amphibian host might present distinctive and complex SAR curve shapes in comparison to macroecological species. Our goal was to fit and compare diverse nonlinear statistical models for the purpose of improving the description of SAR trends for host-specific microbiota and providing adequate ecological explanations for the complexity of microbial SARs. Finally, there are typically three types of amphibians, aquatic, terrestrial and arboreal, and the composition and structure of the skin microflora of amphibian hosts from different habitat types were also expected to differ.

## RESULTS

### Skin microbial diversity of amphibians.

We collected a total of 352 specimens of 10 amphibian host species in 10 transects, the number of specimens and locations of which are shown in detail in Table 1. The most abundant species was *Fejervarya multistriata*, with 118 specimens, and the least abundant species was *Quasipaa spinosa*, with 12 specimens. Most of these 10 species prefer terrestrial and aquatic habitats, except for the species *Polypedates megacephalus*, which is arboreal or tree-dwelling (see Table S1 in the supplemental material). After sequencing and screening, a total of 44,387 microbial operational taxonomic units (OTUs) were obtained from the skin samples of all 352 amphibian specimens. All subsequent analyses were conducted on these microbial OTUs.

**TABLE 1:** Sampling transect information and number of specimens and species collected in the rural Chengdu region of SW China

Transect ID	Longitude	Latitude	Host species recorded	Total no. of specimens
1	104.2818	30.52729	*Pelophylax nigromaculatus*, *Quasipaa spinosa*, *Fejervarya multistriata*, *Hylarana guentheri*, *Bufo gargarizans*	26
2	104.3245	30.41945	*Pelophylax nigromaculatus*, *Microhyla fissipes*, *Fejervarya multistriata*, *Hylarana guentheri*, *Bufo gargarizans*	44
3	104.0531	30.16083	*Pelophylax nigromaculatus*, *Microhyla fissipes*, *Fejervarya multistriata*, *Hylarana guentheri*, *Bufo gargarizans*	36
4	103.9406	29.81665	*Pelophylax nigromaculatus*, *Microhyla fissipes*, *Fejervarya multistriata*, *Hylarana guentheri*, *Bufo gargarizans*	44
5	103.6009	30.02454	*Pelophylax nigromaculatus*, *Microhyla fissipes*, *Fejervarya multistriata*, *Hylarana guentheri*, *Rana chensinensis*, *Bufo gargarizans*	39
6	103.5333	30.66782	*Pelophylax nigromaculatus*, *Quasipaa spinosa*, *Fejervarya multistriata*, *Bufo gargarizans*, *Polypedates megacephalus*	34
7	103.3635	30.62526	*Quasipaa spinosa*, *Fejervarya multistriata*, *Rana chensinensis*, *Bufo gargarizans*, *Odorrana schmackeri*, *Polypedates megacephalus*	31
8	103.2103	30.18856	*Pelophylax nigromaculatus*, *Microhyla fissipes*, *Fejervarya multistriata*, *Rana chensinensis*, *Bufo gargarizans*, *Polypedates megacephalus*	50
9	103.2596	30.18806	*Bufo gargarizans*, *Odorrana schmackeri*, *Amolops mantzorum*	31
10	103.2365	29.7958	*Pelophylax nigromaculatus*, *Fejervarya multistriata*, *Bufo gargarizans*, *Polypedates megacephalus*, *Odorrana schmackeri*	23

**FIG 1:**
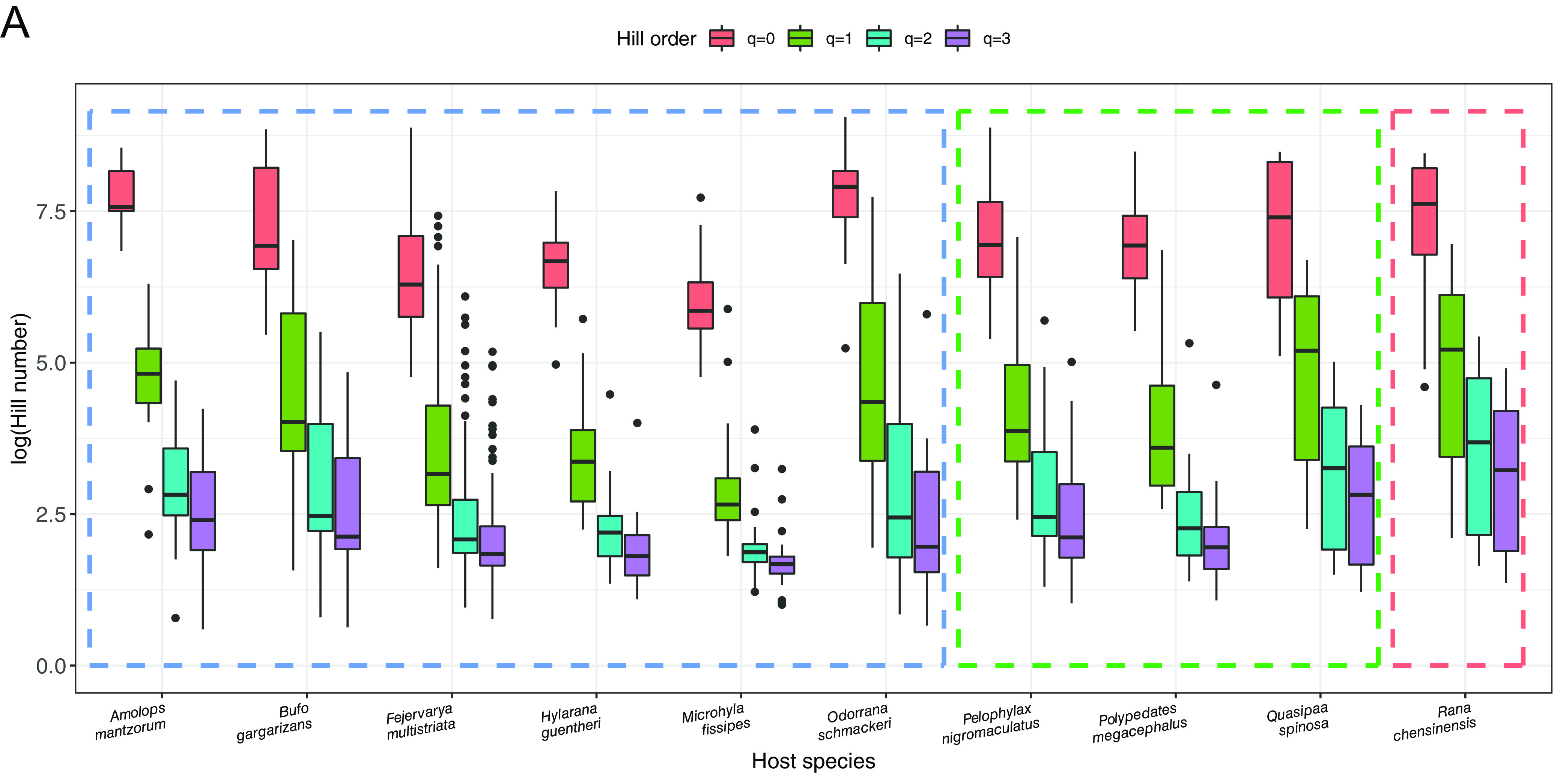

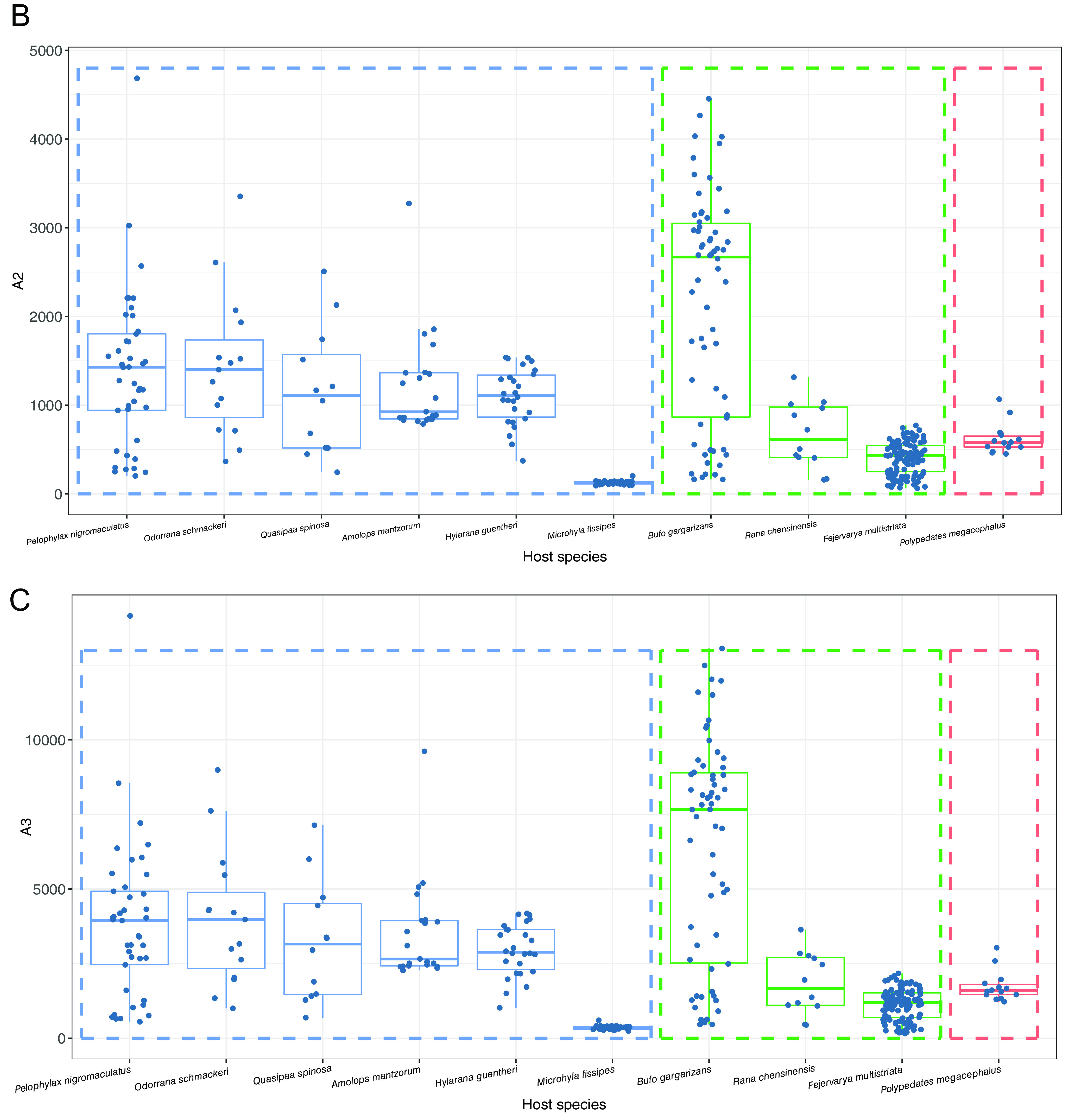
Dispersion of symbiotic microbial diversity in terms of Hill number and the supporting skin surface area sizes for different host species. (A) Dispersion of microbial diversity for different Hill number orders (*q* = 0, 1, 2, 3) from different specimens of host amphibian specie. (B) Dispersion of 2D (i.e., A2) skin area sizes calculated for specimens of varied host species. (C) Dispersion of 3D skin areas (i.e., A3) calculated for specimens of varied host species. In all three plots, dashed boxes are used to indicate the habitat preferences of different host species: blue-dashed box, aquatic; green-dashed box-terrestrial; and red-dashed box, arboreal.

Microbial diversity measured using Hill number was calculated for four orders (*q* was equal to 0, 1, 2, and 3) at the species level (Fig. 1A). In detail, microbial richness (equal to Hill number when *q* = 0) was highest in *Odorrana schmackeri* and *Rana chensinensis* and lowest in *Microhyla fissipes*, and the exponential form of the Shannon index (equal to Hill number when *q* = 1) and Simpson index (equal to Hill number when *q* = 2) was highest in *Rana chensinensis*. Different groups of habitat-preferring amphibian hosts did not show remarkable differences in the Hill numbers for skin microbiota, as indicated by the corresponding Kruskal-Wallis tests (these results were not shown in the figures): *P* = 0.2913, 0.9895, 0.6119, and 0.4685 for microbial diversity with order *q* = 0, 1, 2, and 3, respectively.

### Skin surface area sizes of amphibian hosts.

By using equations 1 and 2, one can see that (Fig. 1B and C) the two-dimensional (2D) surface area sizes of these 10 amphibian host specimens can range from 60 to 5,200 cm^2^, while the 3D surface area sizes ranged from 150 cm^3^ to 15,500 cm^3^ (Fig. 1B and C). The area sizes calculated for each of the two spatial dimensions differed remarkably in value when comparing distinct species. However, body surface area sizes for a specific species were similar when comparing the values for the two spatial dimensions (Fig. 1B versus Fig. 1C). Finally, different habitat-dwelling amphibian hosts (based on the habitat classification of host species in Table S1) did not have remarkable differences in skin area sizes, as indicated by the corresponding Kruskal-Wallis tests: *P* = 0.05989 for 2D skin area measurement and *P* = 0.06605 for 3D skin area measurement (these test results are not shown in the figures).

**FIG 2:**
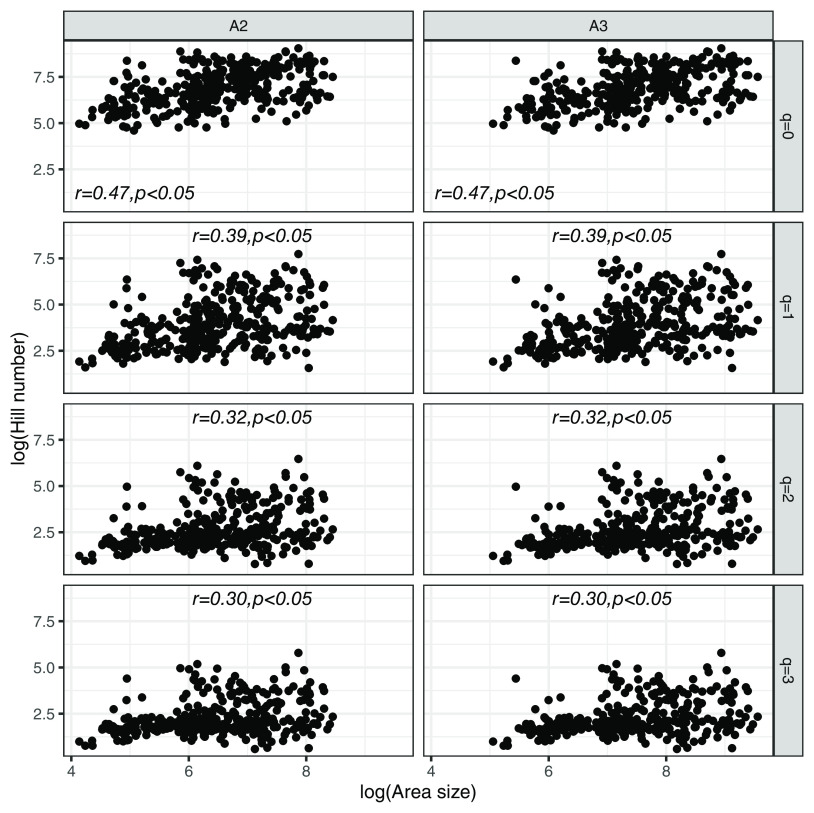
Overall relationship (inside texts correlate values with significant levels) between symbiotic microbial diversity (measured using the order of Hill number [*q* = 0, 1, 2, 3]) and skin area sizes over different host species. For detailed fitting of different SAR models in different host species, please refer to Fig. S3 to S12 in the supporting materials.

The overall relationship between skin area size and microbial diversity in terms of Hill’s number across different host species suggested that (Fig. 2), increasing skin area surface size of host species in general will increase the diversity of symbiotic microbiota. The increasing trends were linear and expected to present significant slopes in all the measurement of skin area and Hill orders (Fig. 2). However, when it came to the host species-specific relationship of skin area and microbial diversity, the stories were totally different (the details are given below).

### Host-specific microbial SARs.

After we calculated the microbial diversity and skin surface area sizes of each amphibian specimen, we fitted different SAR models to find the best ones; the fitting results are shown in detail in Fig. S3 to S12 in the supplemental material. In contrast to the crossing-host results showing that microbial diversity increased as skin area increased (Fig. 2), the host-specific symbiotic microbial SAR curves presented two additional distinctive trends: (i) corresponding to the PLEC (power-law with experimental cutoff) model, as the area increases, the microbial diversity first rises and gradually decreases after reaching maximum accrual diversity (MaxAD), and (ii) corresponding to the PLIEC (power-law with inverse experimental cutoff) model, as the area increases, the microbial diversity decreases from a certain point and starts to rise after reaching minimum accrual diversity (MinAD). Also, the one-breaking point model (BR) model generally describes the turning point, regardless of the changing trend of the microbial SAR model, and can be used to find both MaxAD and MinAD. For example, in supporting materials (see Fig. S7), we show that the BR model can identify MinAD for *Polypedates megacephalus*, while Fig. S11 showed that the BR model can help detect MaxAD for *Rana chensinensis*.

**FIG 3:**
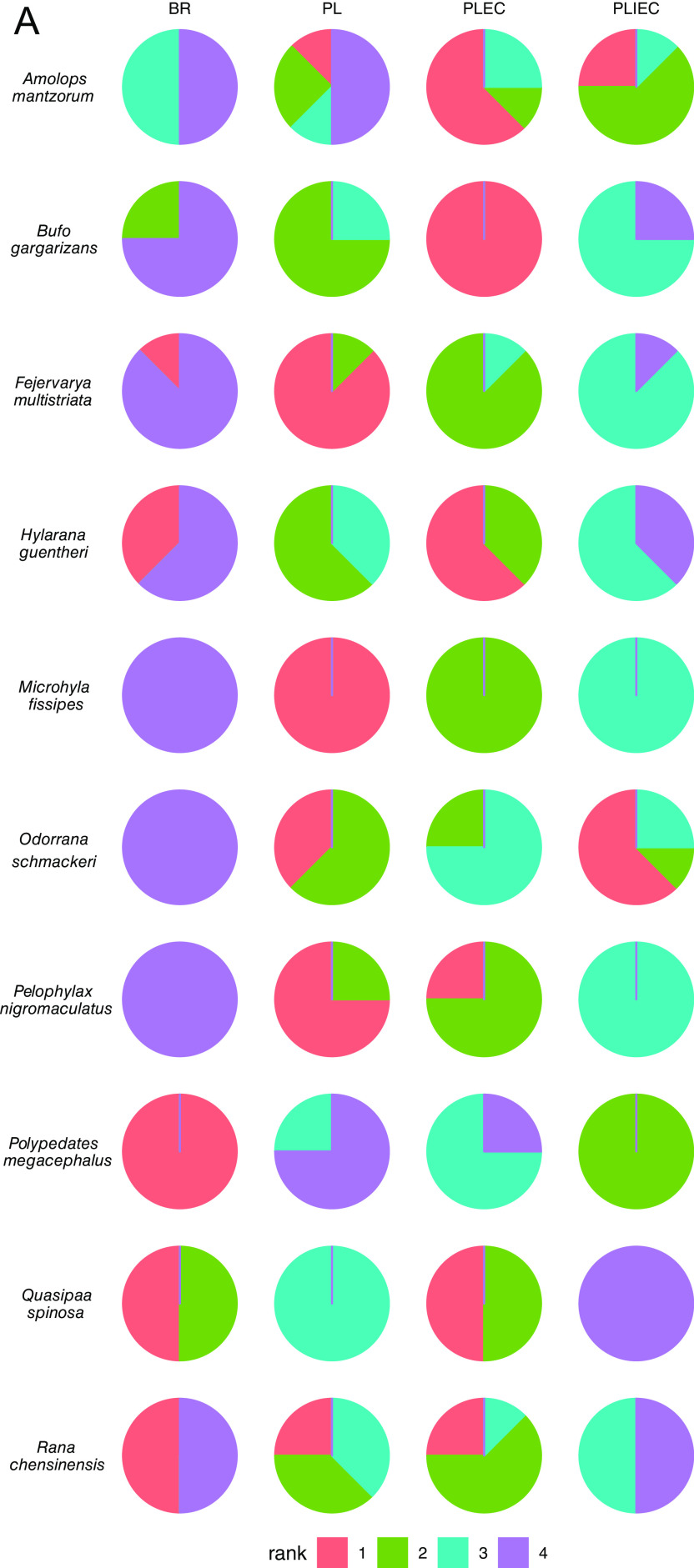

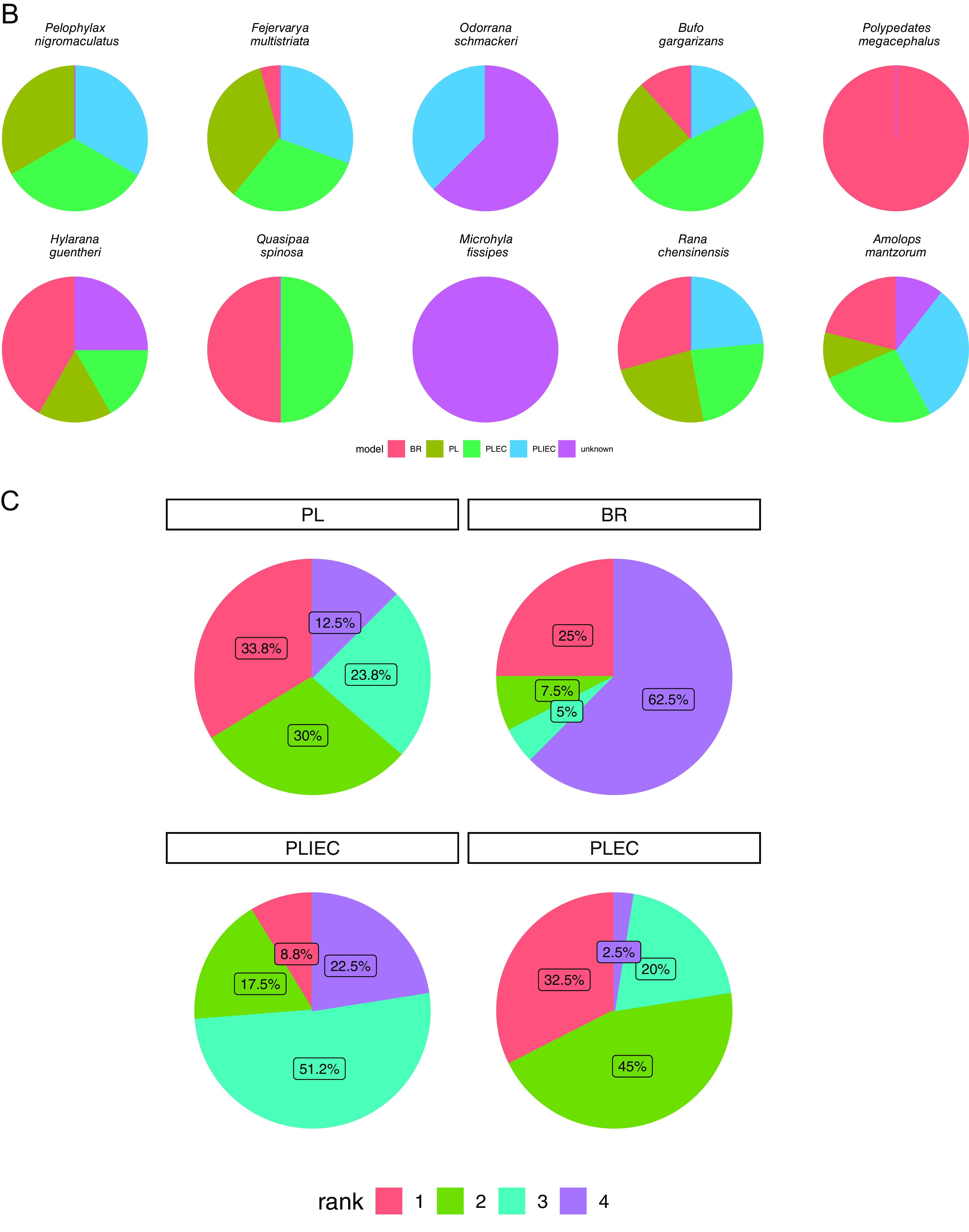

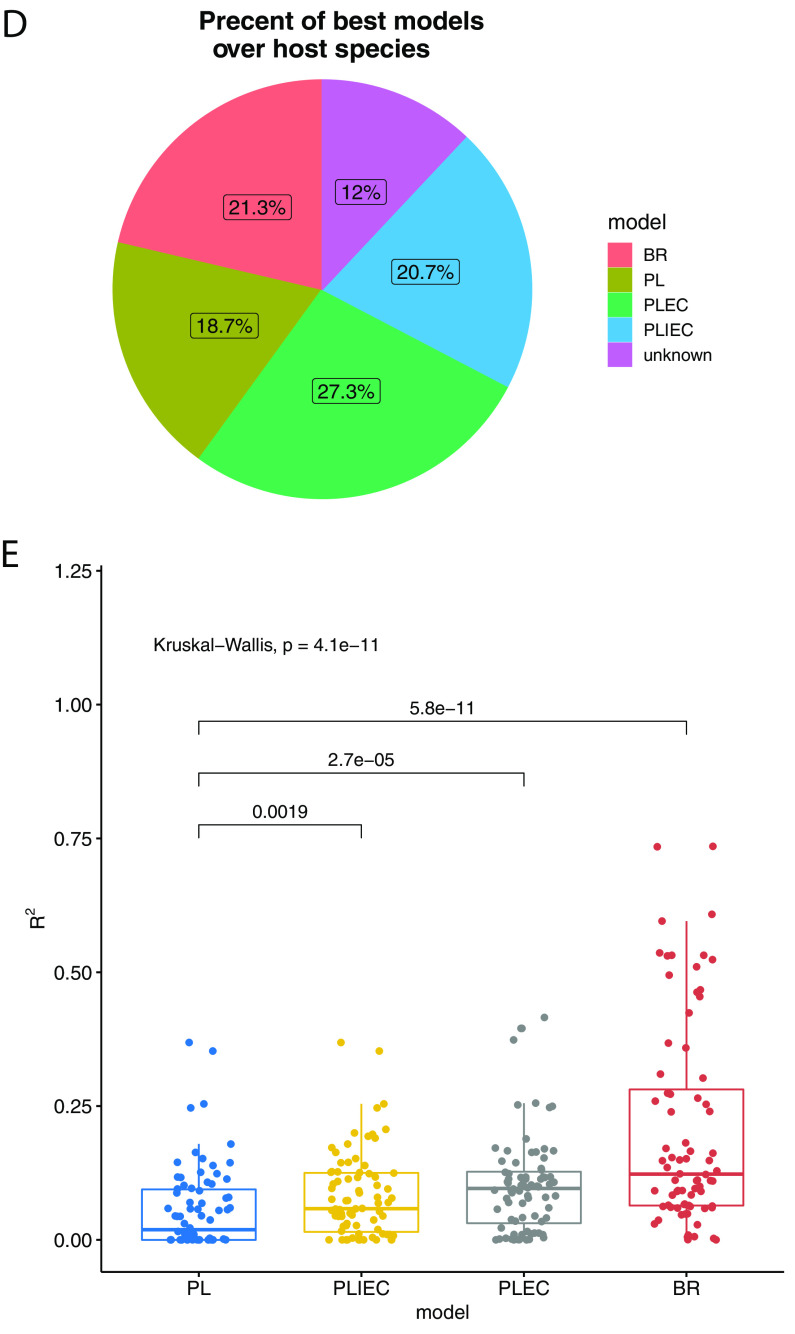
Pie charts showing the summary statistics comparing the performance of symbiotic microbial SAR models that were applied to different host species. The percentage pie chart ranks are based on the AIC weights of different symbiotic microbial SAR models when applied to each of the host species (A) and percentage pie chart of equivalently optimal models selected for describing skin microbial taxa over specimens for each of the 10 host amphibian species (B). (C and D) Overall summary statistics for the AIC weight-based rank comparison and equally optimal model-based comparison of different SAR models over different host species, respectively. (E) Comparison of the fitting powers (in terms of *R*^2^) of different models when applied to the skin symbiotic microbial diversity of host amphibian species.

In general, symbiotic microbial SARs from different amphibian species were found to favor one, some, or all of the three trends described above, as indicated, for example, by both AIC weight rank analysis and/or optimal model selection procedure (Fig. 3A and B). Thus, *Polypedates megacephalus* only favored BR model, while *Quasipaa spinosa* preferred two models (BR and PLEC). Finally, *Hylarana guentheri* favored three models (power-law [PL], BR, and PLEC) (Fig. 3A and B). Notably, no models could fit the skin area-microbial diversity relationships well for host species *Microhyla fissipes*. Even though the PL model was ranked first for the microbial data sets of this host species, the fitting powers (in terms of *R*^2^) actually were very low (<0.05), and therefore it was considered to be inadequate based on the criteria defined in our optimal model selection procedure.

As expected, two different ways of assessing model performance (i.e., AIC weight versus optimal model selection) generated very similar results at host species level (Fig. 3A and B). For example, the BR model was ranked with the highest score in species *Polypedates megacephalus*, but was also exclusively favored in AIC weight rank analysis, regardless how skin area is measured and how microbial diversity is calculated (Fig. 3A and B). As another example, the other three models (PL, PLEC, and PLIEC) were favored by symbiotic microbiota of *Pelophylax nigromaculatus* in most cases in optimal model selection procedure, and PLEC and PL were also ranked very high in the AIC weight rank analysis.

### Crossing-species microbial SAR model comparison.

In overall, both PL and PLEC models can be ranked first in some microbial diversity data sets for six host species (Fig. 3C) in AIC weight rank analysis. However, the PLEC model was the one with highest preference in optimal model selection analysis (Fig. 3D), since it can be selected in seven host species (Fig. 3B). The BR and PLIEC models could also be selected with preference in some species. For example, as mentioned above, *Polypedates megacephalus* favored the BR model exclusively (Fig. 3A and B). When taking both AIC weight rank results and optimal model selection procedure results into account, one can conclude that PLEC was the best-fit model with highest selection frequency, followed by the classical PL model. BR and PLIEC models had their unique contributions, particularly in some specific host species.

However, when it came to the fitting powers of different models over different host species (Fig. 3E), it was found that, although PL model was usually selected with very high popularity in the procedure of model comparison and selection (Fig. 3A to C), its overall fitting power actually was the lowest (Fig. 3D). Its *R*^2^ was statistically lower than the other three advanced models, as indicated the pairwise Wilcoxon’s test and the overall Kruskal-Wallis test (Fig. 3E). This implies that simple statistical models could be applicable in most cases, it is difficult to link the underpinning ecological mechanisms. In contrast, PLEC, PLIEC, and BR models had more abundant ecological interpretations. The changing trends before and after the key threshold (i.e., MaxAD, MinAD, or inflection point) could be associated with ecological processes and deserve further discussions.

### Skin area thresholds for microbial SARs.

**FIG 4:**
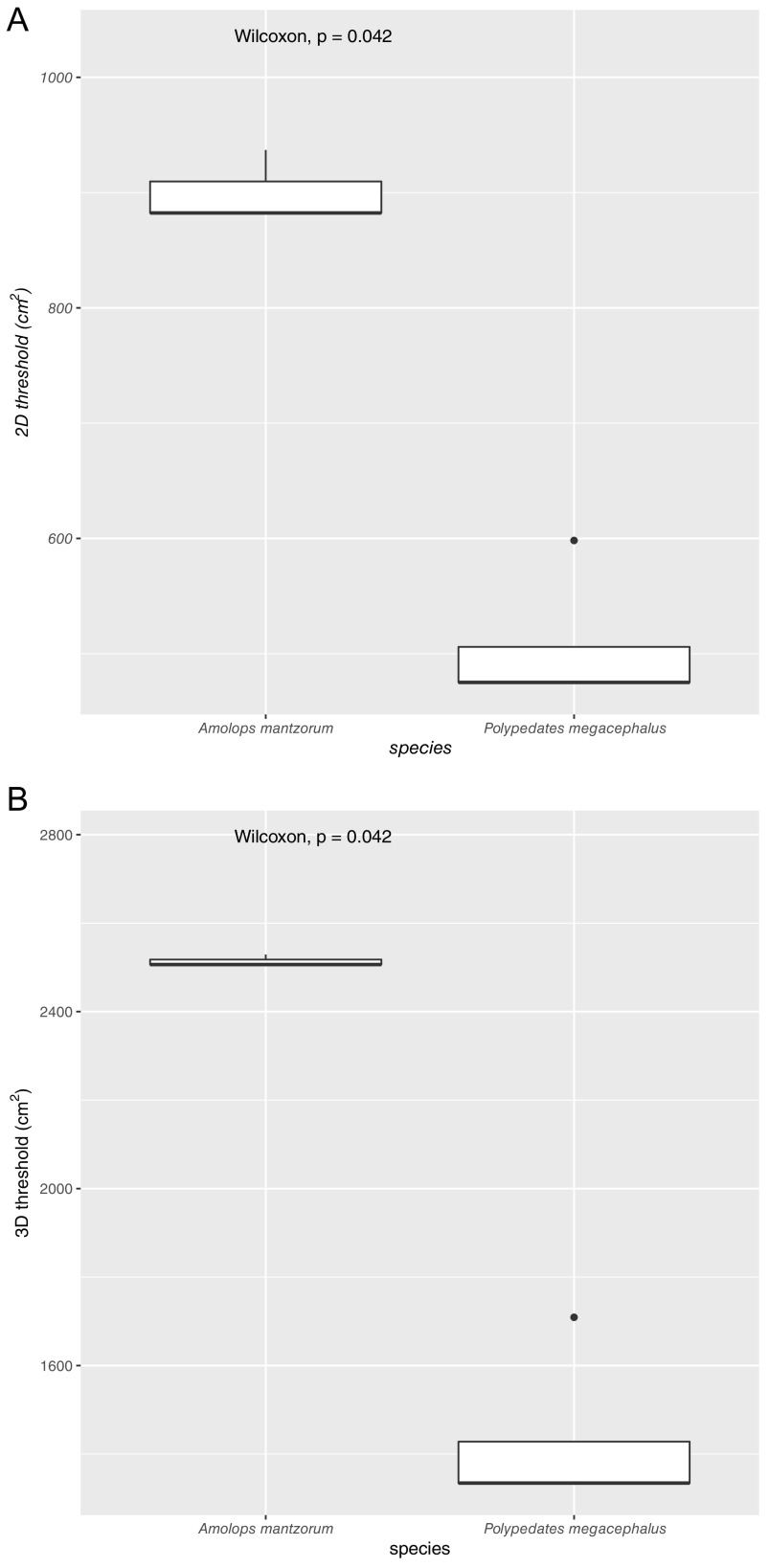

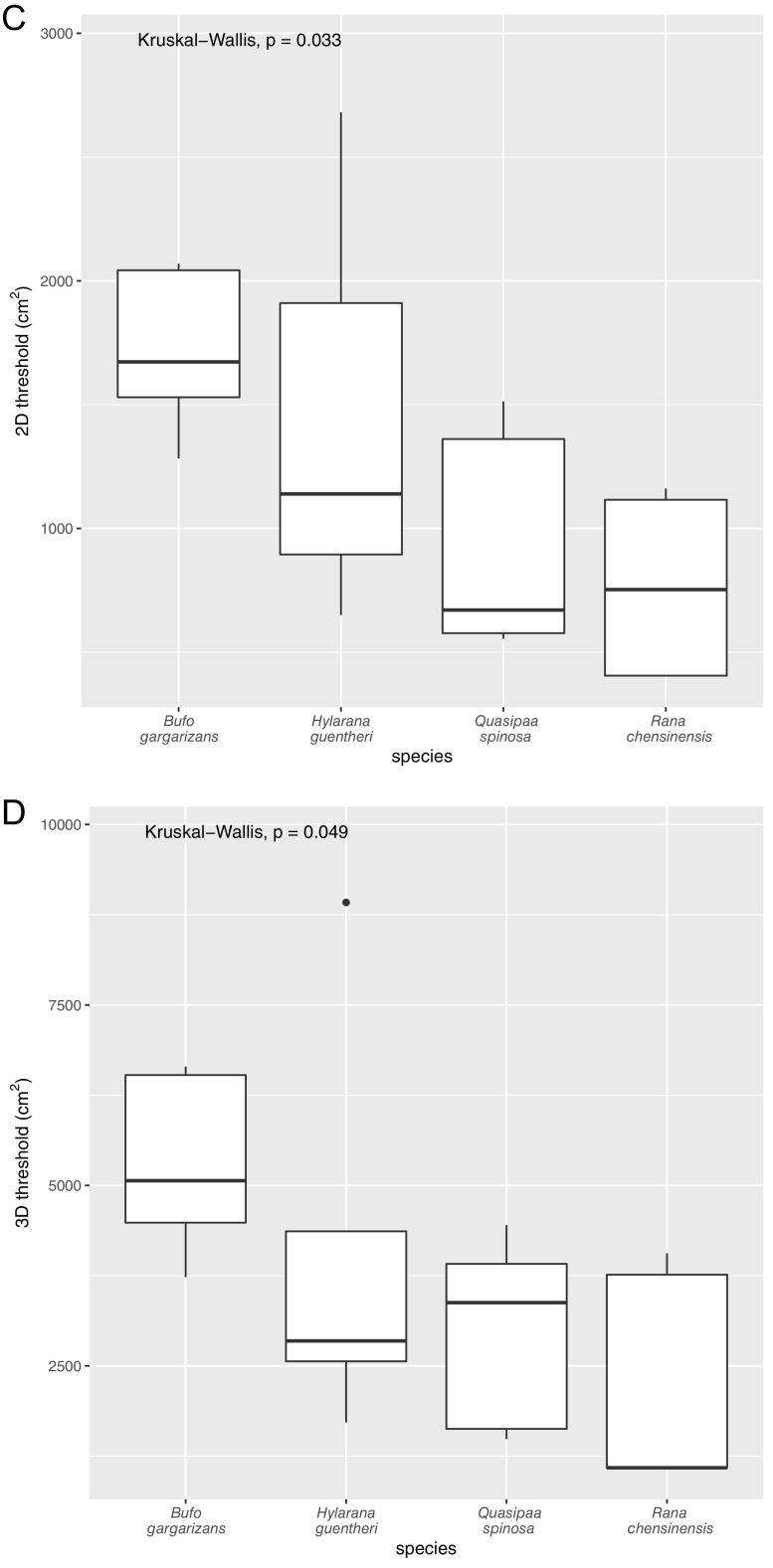

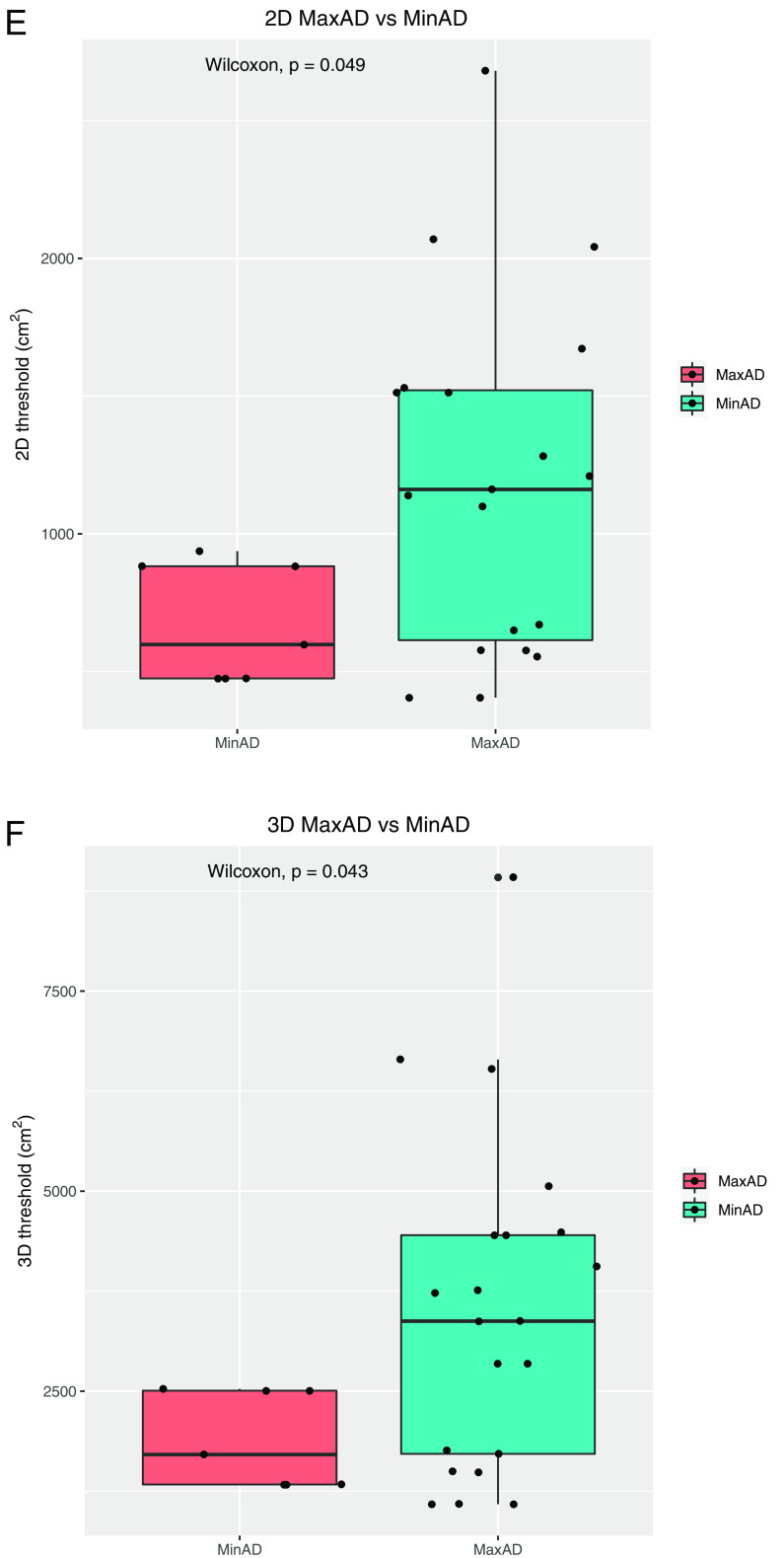
Summary and comparison of skin area thresholds based on the fitting results of three SAR models (PLEC, PLIEC, and BR). (A and B) Comparisons of 2D and 3D skin area thresholds for obtaining MinAD of host species (only those with MinAD available are presented). (C and D) Comparison of 2D and 3D skin area thresholds for obtaining MaxAD of host species (only those with MaxAD available are presented). (E and F) 2D skin area threshold comparison for obtaining MaxAD versus MinAD across different host species (if available) (E) and 3D skin MaxAD versus MinAD area size comparison across different host species (if available) (F).

At the species level, we found some hosts can allow the occurrence of MaxAD or MinAD. For example, two host species, *Amolops mantzorum* and *Polypedates megacephalus*, were found to allow the emergence of MinAD in their specimens (Fig. 4A and B). As a comparison, four host species *Bufo gargarizans*, *Hylarana guentheri*, *Quasipaa spinosa*, and *Rana chensinensis* could show MaxAD in their specimens (Fig. 4C and D). Notably, skin area size thresholds for reaching MinAD or MaxAD were statistically different among these species, as indicated by the respective Wilcoxon test and Kruskal-Wallis test. Finally, at the community level, by lumping the results of all host species, no matter whether 2D or 3D cases, host skin surface area sizes for obtaining MinAD versus MaxAD were statistically significantly different, i.e., skin area sizes where MinAD was reached were usually lower than those where MaxAD was obtained (Fig. 4E and F).

The results further indicated that, in general (Fig. 4E and F), for a specific host amphibian species, if its body surface area size is ~600 cm^2^ (for 2D measurement) or ~1,250 cm^2^ (for 3D measurement), then it is likely to observe MinAD of skin-borne microbiota. As a comparison, if its body area size is ~1,400 cm^2^ (for 3D measurement) or ~3,500 cm^2^ (for 3D measurement), skin-borne microbiota would reach MaxAD. Increasing body size would not increase but likely decrease the diversity of skin-borne microbe. Of course, different amphibian host species would have their own skin area thresholds of obtaining MinAD and MaxAD. This can be a quite interesting question deserving further investigation.

## DISCUSSION

According to the results, in many cases, microbial diversity (calculated in terms of Hill number) will reach MaxAD or MinAD, which might not be frequently seen in previous studies on SARs of animals and plants (17). Further, through extensive model selection and comparison at the host-species and community levels, we found that PLEC model was the most superior in fitting the symbiotic microbial SAR data sets (Fig. 3). As a comparison, because of simplicity, the PL model was also selected as one of the best-fit models with the second highest preference in AIC weight rank analysis. However, the fitting performance of the classical PL model was in general the lowest and significantly lower than the other three SAR models. This implies that the introduction of biologically complex SAR models, such as the PLEC, PLIEC, and BL models advocated in the present study, are useful to help microbial biologists to interpret the ecological mechanisms and processes driving the observed SAR curve shape patterns.

Based on our literature review, we believe that the reasons for this discrepancy could be multifaceted.

First, Preston (18) proposed that the sampling method can be divided into accumulated area and isolated area, which is classified as the second type in our study. Tjørve and Turner (19) then suggested that isolated islands do not increase in diversity as they increase in size. They proposed that due to the boundary effect, species diversity will first gradually increase with the increase in the area within the isolated boundary, but there will be an inflection point somewhere, and the diversity after the inflection point will not continue to increase with the increase in the area. Perhaps due to the nature of microorganisms, microbial diversity here does not fluctuate around the inflection point but begins to decline after reaching the inflection point. We believe that one of the reasons that the microbial SAR trend can show a PLEC-type curve is that, initially, the microbial diversity increases with the increase in the sampling area, but it does not increase infinitely. Due to the interaction between the sampling saturation effect and the interactions (competition in microbial communities), the microbial diversity level reaches MaxAD. Afterward, due to factors such as random drift, the microbial diversity will not fluctuate around this point but will decrease as the area increases.

Second, the small-island effect (SIE), i.e., the pattern that species richness on islands below a certain area threshold can vary independently of area, has become recognized in island biogeography and biodiversity research (20). When the microbial diversity-area relationship can be expressed as the PLIEC function, similar to the SIE when the skin surface area of host species is less than a threshold value, the microbial diversity does not increase with the area but varies independently (declining) of the skin area of hosts. The reason for this, we suspect, is that when the habitat area size is small, the space available for the various microbes to survive is not sufficient for them to form a stable community.

Third, the bare and moist characteristics of amphibian skin make it prone to microbial infection. To resist the invasion of pathogenic microorganisms, amphibians form an immune system with antimicrobial peptides as the main defense effector during long-term natural evolution (21, 22). In fact, antimicrobial peptides (AMPs) are widely distributed in animals, plants, and microorganisms and are pivotal weapons for hosts to resist bacteria, fungi, viruses, and protozoa (23, 24). AMPs represent ancient and effective natural defense substances in evolution (25). We speculate that the larger individual amphibians are, the greater the number of antimicrobial peptides they can secrete, and the stronger the selection effect on commensal microorganisms, retaining only probiotics. Therefore, when the surface area or volume of the host reaches a certain threshold, the microbial diversity decreases rather than rises, and the microbial DAR can be expressed as the PLEC function.

Fourth, due to multihost and multisite interactive effects, i.e., we believe that the lumping of multihost species and multisite specimens would create huge heterogeneity when analyzing microbial SARs from the specimen skins. Furthermore, different host species usually prefer different abiotic conditions, and skin microbial taxa can be environmentally influenced in different sampling locations and habitats. Given that microbial community structure and diversity are expected to be sensitive to environmental and host changes, our present multihost analyses could create diverse and complex skin microbial SARs that might be less frequently found in macroorganism communities.

In summary, through multihost analyses, this study showed that microbial SARs are more complex than expected, and their nonlinearity effect may be attributed to various ecological mechanisms. For further research, it would be of value to disentangle the relative importance of different underpinning mechanisms in influencing the SAR nonlinearity pattern. Given that high-throughput sequencing techniques could generate unprecedented microbial diversity data, it would be of equal interest to introduce and develop advanced statistical methods to explore the microbial community ecology and diversity patterns for the purpose of discovering novel microbial diversity and distributional patterns that might rarely be observed in macroecological studies.

## MATERIALS AND METHODS

### Sample collection.

In this study, amphibian specimens were collected from 10 transect locations in the rural areas of Chengdu city, Sichuan Province, SW China (Table 1). To avoid contaminating the amphibian skin, we wore sterile gloves to collect samples. For consistent sampling standards, sterile swabs that had no germicidal effects on the microbes were used to rub the head, back, side, and abdomen of each animal three times. The swabs were then transferred to 2-mL aseptic centrifuge tubes and stored at −80°C until they were extracted for DNA analysis. Our experiments were approved by the Institution of Animal Care and the Ethics Committee of Chengdu Institute of Biology, Chinese Academy of Science(permit no. CIBDWLL2022008).

### Microbial analyses.

Total community genomic DNA extraction was performed using an E.Z.N.A. Soil DNA kit (Omega, USA) according to the manufacturer’s instructions. We measured the concentration of the DNA using Qubit 2.0 (Life, USA) to ensure that adequate amounts of high-quality genomic DNA had been extracted.

PCR was started immediately after the DNA was extracted. The 16S rRNA V3-V4 amplicon was amplified using KAPA HiFi Hot Start Ready Mix (2×; TaKaRa Bio, Inc., Japan). Two universal bacterial 16S rRNA gene amplicon PCR primers (PAGE purified) were used: the amplicon PCR forward primer (CCTACGGGNGGCWGCAG) and amplicon PCR reverse primer (GACTACHVGGGTATCTAATCC). The ratio of the reaction system (total, 30 μL) was as follows: microbial DNA (10 ng/μL), 2 μL; amplicon PCR forward primer (10 μM), 1 μL; amplicon PCR reverse primer (10 μM), 1 μL; 2× KAPA HiFi Hot Start Ready Mix, 15 μL; and distilled water, 30 μL. The plate was sealed, and PCR was performed in a thermal instrument (Applied Biosystems 9700, USA) using the following program: 1 cycle of denaturing at 95°C for 3 min; 5 cycles of denaturing at 95°C for 30 s, annealing at 45°C for 30 s, and elongation at 72°C for 30 s; and then 20 cycles of denaturing at 95°C for 30 s, annealing at 55°C for 30 s, elongation at 72°C for 30 s, followed by a final extension at 72°C for 5 min. The PCR products were checked using electrophoresis in 1% (wt/vol) agarose gels in TBE buffer (Tris, boric acid, EDTA) stained with ethidium bromide (EB) and visualized under UV light.

We used AMPure XP beads to purify the free primers and primer dimer species in the amplicon product. Samples were delivered to Sangon Biotech (Shanghai) for library construction using a universal Illumina adaptor and index. Before sequencing, the DNA concentration of each PCR product was determined using a Qubit 2.0 Green double-stranded DNA assay, which was quality controlled using a bioanalyzer (Agilent 2100, USA). Depending on coverage needs, all libraries can be pooled for one run. The amplicons from each reaction mixture were pooled in equimolar ratios based on their concentration. Sequencing was performed using the Illumina MiSeq system (Illumina MiSeq, USA) according to the manufacturer’s instructions.

After sequencing, the data were manipulated as follows: (i) PEAR software was used to assemble the two short Illumina readings (v0.9.6, https://cme.h-its.org/exelixis/web/software/pear/) according to the overlap, and fastq files were processed to generate individual fasta and qual files, which could then be analyzed by standard methods. (ii) Sequences containing ambiguous bases and any longer than 480 bp were dislodged, and those with a maximum homopolymer length of 6 bp were allowed (26). Furthermore, sequences that were shorter than 200 bp were removed. (iii) All identical sequences were merged into one. (iv) Sequences were aligned according to a customized reference database. (v) The completeness of the index and the adaptor was checked, and all of the index and the adaptor sequence were removed. (vi) Noise was removed using the Precluster tool. Chimeras were detected by using Chimera UCHIME (https://mothur.org/wiki/chimera.uchime/). All the software was in the mothur package. We submitted the effective sequences of each sample to the RDP Classifier (http://rdp.cme.msu.edu/classifier/classifier.jsp) again to identify bacterial sequences. Finally, all effective bacterial sequences without primers were clustered under 97% similarity to obtain the microbial OTU table for downstream analysis (27).

### Skin area measurement.

We collected the morphological data of the samples, including body length, head width, and head length. These trait values were used to construct equivalent two-dimensional and three-dimensional models. As we can see from Fig. S10, first, we perform a 2D transformation using the head width of the amphibian specimen as the base and the head length as the height to construct a triangle. Second, we used the head width as the base and the body length as the height to construct a rectangle; at this point, the sum of these two geometric objects forms the skin surface of the frog specimen.

Assuming that head length is denoted as *a*, head width is denoted as *b*, and body length is denoted as *h*, through 2D geometric transformation, we obtained the 2D skin surface area size (A2) by (28):

(1) $$S1=\frac{\mathrm{ab}}{2}+\;b\times (h-a)$$

Then, we perform a 3D transformation, using the head width as the base diameter and the head length as the height to construct a cone and then using the head width as the base diameter and body length as the height to construct a cylinder. Thus, the external surface area of two geometries can be used to represent the skin surface area size of an amphibian host organism. The 3D skin surface area size (A3) thus can be computed as follows (28):

(2) $$S2=\frac{\pi b\sqrt{a^{2}\;+\frac{b^{2}}{4}}}{2}\;+\pi b\left(h-a\right)\;+\frac{\pi b^{2}}{4}$$

Each of the two calculation formulas above is applied to specimens of different amphibian species (*Anura*).

### Microbial diversity measurement.

Two components comprise the community: one is the number of species, and the other is the relative abundance of distinct species. Traditionally, the SAR has been limited to the relationship between species richness (number of species) and area (space), while species abundance is usually neglected. The OTU table generated from 16S rRNA amplicons and bioinformatics pipelines contains not only information about species richness (the number of species) but also the abundance of each species simultaneously. To capture both species richness and abundance information, we adopted Hill numbers as diversity measures. Hill numbers are a mathematically unified family of diversity indices that incorporate relative abundance information and species richness information, which encompass the group of diversity measures that quantify diversity in units of equivalent numbers of equally abundant OTUs or species (29, 30). In addition, Hill numbers offer the most appropriate measures for alpha diversity and multiplicative beta diversity partitioning. Because of the generality and flexibility in controlling the effects of rare taxa in biodiversity measures, the Hill number appeared to represent excellent framework for microbial diversity studies.

Hill number (29, 30) was proposed as a unified diversity concept by defining biodiversity as a reciprocal mean proportional abundance and differently weighing taxa based on their abundances as follows:

(3) Dq=(∑i=1Spiq)11−q

where *s* is the number of species, *p_i_* is the relative abundance of the *i*th species, and *q* is called the order of diversity, which determines its sensitivity to species frequencies. The measure ^0^*D* corresponds to species richness, and ^2^*D* corresponds to the inverse Simpson concentration, giving roughly the number of “very abundant” species in a community. The measure is undefined when *q* = 1, but the limit as *q* approaches unity exists and equals the exponential of Shannon entropy, measuring the number of “common” (or “typical”) species in a community. In other words, the detailed mathematical forms of the three orders of the Hill number are given as:

(4) Dq=(∑i=1Spiq)11−q={S, q=0exp{−∑i=1Spilog(pi)}, q=11∑i=1Spi2, q=2

We believed that Hill numbers should follow the same or similar trends as traditional SARs because *^q^D*(*q* ≤ 2) values are strongly influenced by species richness (i.e., *S*). After all PCR products were sequenced, we calculated Hill indices of order 0 to 3 according to the OTU table as indicators of skin microbial diversity.

### Microbial SARs.

After we obtained data on skin area and microbial diversity, we investigated the microbial DAR. Conventional SARs have multiple functional forms; however, there is no clear biological foundation to give preference to these particular models (31), and the best-fitting model for a particular species-area curve can only be determined empirically (32). To study the changing law of microbial diversity with area size, we fitted the best well-known power law (PL) model. We also included PL with exponential cutoff (PLEC) and PL with inverse exponential cutoff (PLIEC) (16, 17). Finally, we also introduced a one-breaking point model (BR) for comparison.

The PLEC model is described as follows:

(5) $$f\left(A\right)=cA^{z}\text{exp}\left(\mathrm{bA}\right)$$

where *f*(*A*) is the diversity measured in the Hill numbers, and *b* < 0 is set in our study. At the beginning, *f*(*A*) increases in response to increasing *A*. However, as the maximal value of exp(*bA*) is 1 when *A* = 0, so when *A* becomes larger, exp(*bA*) will decrease and become smaller (being close to 0). Accordingly, *f*(*A*) will decrease in response to increasing *A*. PLEC is likely to reach a maximum when its derivative equals zero, that is,

(6) $$(\mathrm{df}(A))/\mathrm{dA}=[cA^{Z}\exp\left(\mathrm{bA}\right)]\prime =0$$

Hence, when

(7) $$A_{\text{max}}=-z/b$$

*f*(*A*) may have a maximum in the following form:

(8) $$\text{Max}\left(f(A)\right)=c{\left(-\frac{z}{b}\right)}^{z}\exp\left(-z\right)=cA_{\text{max}}^{z}\exp\left(-z\right)$$

may have a maximum in the following form. Equations 7 and 8 were utilized to predict the maximal accrual diversity (MaxAD) of the microbiome (16).

The PLIEC model has the following form:

(9) $$f(A)=cA^{z}\text{exp}(d/A)$$

In our study, we assume the following constraint as *d* > 0. In contrast to the PLEC model, when *A* becomes larger, will increase from 0 and become larger and closer to 1. In contrast, *cA^z^* will always increase. As such, there can be a tradeoff between *cA^z^* and exp(*d/A*), and this might obtain a minimum when its derivative equals zero, that is,

(10) $$\frac{\mathrm{df}(A)}{\mathrm{dA}}=\mathrm{cz}A^{z-1}\text{exp}(d/A)-cA^{z}\frac{d}{A^{2}}\text{exp}(d/A)=0$$

(11) $$zA^{-1}\text{exp}(d/A)-\frac{d}{A^{2}}\text{exp}(d/A)=0$$

When *A* → Inf, exp(*d/A*) → 1 or when *A* = *A*_min_ = *d/z*, equation 6 holds, but it is biologically impractical for *A* → Inf. Corresponding to the MaxD of the PLEC model when:

(12) $$A=A_{\min}=d/z$$

*f*(*A*) may have a minimum in the following form:

(13) $$\text{Min}\left(f(A)\right)=c{\left(\frac{z}{d}\right)}^{z}\exp\left(z\right)=cA_{\min}^{z}\exp\left(z\right)$$

Equations 12 and 13 can be utilized to predict the minimum accrual diversity (MinAD) of the microbiome. Note that it is also biologically impractical when *A* → Inf and *A* > 0, *A*_min_ < 0. Interestingly, in addition to MinAD, this model can present a positive inflection point (Table 2; see also Fig. S1), detailed derivation can be found in the supporting materials.

**TABLE 2:**
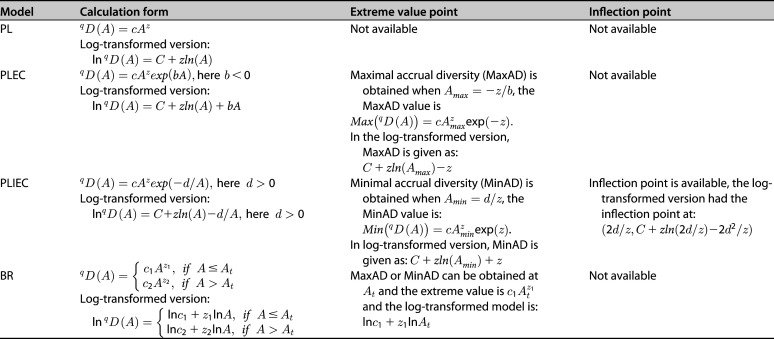
Statistical features of microbial SAR models used and compared in the present study

Finally, the one-breaking point model (BR) can be formulated as follows:

(14) ln⁡Dq(A)={ln⁡c1+z1ln⁡A, if A ≤ Atln⁡c2+z2ln⁡A, if A>At

*A_t_* is the area threshold at which the change point of the microbial diversity curve exists. Note that BR model can also be used to obtain MinAD or MaxAD, depending on change of linear trends before and after the breaking point at *A_t_*. The mathematical criteria of obtaining MinAD and MaxAD using BR model and the visual demonstration can be found in the supporting text and Fig. S2 of the supporting materials, respectively.

### Model comparison and selection.

Since skin microbes play a vital role in the environmental adaptability of amphibians, the skin microbes of samples are expected to be different significantly among host species (33, 34). Therefore, microbial diversity data sets for fitting alternative SAR models were constructed based on the Hill number and skin area sizes of each specimen of different host species. In detail, for each specimen of a specific host species, eight microbial diversity data sets can be constructed based on the Hill number (*q* = 0, 1, 2, and 3) and sample skin area sizes (A2 and A3). We utilized two methods for conducting model comparison and selection. The first method is the rank of AIC weight of different SAR models, while the second method is the direct identification of equivalently best optimal models. We believe that these two complementary methods could increase the supporting strength of the candidate appropriate models.

In detail, for a specific data set, such as in 2D for Hill number *q* = 0, for an individual skin specimen from a specific species *Polypedates megacephalus*, we fit different SAR models (Table 2) to the associated symbiotic microbial diversity data. Then, through model fitting, we obtained the Akaike information criterion (AIC) (35) of each fitted SAR model that had been applied and compared, the calculation of which is given as:

(15) $${\text{AIC}}_{i}=-2\log L_{i}\;+\;2V_{i}$$

where *L_i_* represents the maximum likelihood function of model *i*, and *V_i_* is the number of parameters of the SAR model *i* (here *i* = PL, PLIEC, PLEC, or BR). AIC rewards model accuracy by maximum likelihood estimation and punishes model complexity by the number of free parameters. Therefore, the model with smaller AIC value was superior. In addition, we obtained the *R*^2^ value of each SAR model for model comparison. In our study, because we used ordinary least-square technique to estimate the parameters, the exact maximum likelihood form for computing *L_i_* is presented in the supporting materials.

When AIC values were obtained, for the AIC weight-based model assessment method, we first find the minimum value of the AIC values of the four compared models when applied to a specific microbial data set; we then subtract the minimum value by the AIC value of each model to get ΔAIC:

(16) $$\Delta_{i}\text{AIC }=\text{ AICi}\;-\;\text{minAIC}$$

The smaller is ΔAIC, the better is the model fitting. Then, an Akaike weight (wAIC) is calculated according to ΔAIC as:

(17) $${\text{w}}_{i}\left(\text{AIC}\right)=\frac{\text{exp}\left\{-\frac{1}{2}\Delta_{i}(\text{AIC})\right\}}{\sum_{k=1}^{K}\text{exp}\left\{-\frac{1}{2}\Delta_{k}(\text{AIC})\right\}}$$

where *K* represents the number of models compared (i.e., =4) for a specific host specimen. As such, for each host species, we compute wAIC for the four compared models for each specimen, we then rank the wAIC for the purpose to evaluate the performance of each model in fitting skin microbial diversity for each species and across all the species use pie charts. The rank is simply a numeric number of 1, 2, 3, or 4. For a specific data set, the model with the highest wAIC is ranked 1, while the model with the lowest wAIC will be ranked as 4.

For optimal model selection procedure for assessing different SAR models, we obtained the best-fit models based on the following criteria. (i) The first best-fit model was selected as the one with the lowest AIC value. (ii) However, when two models (one of them was the first selected model) have AIC value differences of <2, then both models are believed to perform equivalently well when fitting microbial datasets. These models thus are called equally best-fit models. (iii) Among these equivalently optimal models, if a model had a fitted power *R*^2^ < 0.05, we will discard it from equally best-fit model list. We believe that a SAR model should have a reasonably fitted power, otherwise, it is ecologically meaningless in prediction and discussion. For a specific dataset or host species, if all the SAR models investigated here do not have fitted powers *R*^2^ > 0.05, then we argue that other unknown SAR models should be included and compared, deserving further investigations.
