# Supplementary material for: Microbial Species-Area Relationships on the Skins of Amphibian Hosts
Source: Microbiol Spectr. 2023 Mar 30;11(3):e01771-22. doi: 10.1128/spectrum.01771-22 (PMC10269671; doi:10.1128/spectrum.01771-22)

Supporting information

Microbial species-area relationships on the skins of amphibian hosts

Derivation of inflection points for different SAR models

By setting $f(A) = ln\ {}^{q}D(A)$, for PLIEC model, we have

$$f''(A) = -z/A^2 + 2d/A^3 = 0$$

Therefore, we have

$$A_{Inflection} = 2d/z$$

Because $d > 0$ and $z > 0$, when $A < A_{Inflection}$, one can observe that,

$$f''(A) = -z/A^2 + 2d/A^3 = \frac{2d - zA}{A^3} > 0$$

Therefore, the curve shape of the PLIEC model is convex in this case.

By comparison, When $A > A_{Inflection}$, one can observe that,

$$f''(A) = -z/A^2 + 2d/A^3 = \frac{2d - zA}{A^3} < 0$$

Therefore, the curve shape of the model is concave under this condition.

It can be concluded that $A_{Inflection} = 2d/z$ is the position where the inflection point of the PLIEC model is located. And accordingly, the associated function value is $C + zln(2d/z) - 2d^2/z$.

By contrast, for PL model and PLEC model, one can see that,

$$f''(A) = -\frac{z}{A^2}$$

It is impossible to find roots for $f''(A)$ unless $A \to \infty$. Therefore, no inflection points are available for these two models.

Finally, for BR model, because it is not continuous at A_t, at the left and right sides of A_t, based on the above discussion with respect to PL model, no inflection points can be found.

We presented some numerical investigations on the inflection point presented by the PLIEC model. To be specific, we varied the parameter z while fixing the other parameters as c=1 and d=0.05 for showing the curve shapes of the PLIEC model and the locations of MinAD and inflection point. When z is very small, the location of inflection point is far distant from the MinAD threshold point. By contrast, when z becomes larger, the location of the inflection and MinAD thershold point tends to be closer (Figure S1).

Criteria for obtaining MinAD and MaxAD using BR model

In log-transformed sense, BR model essentially was composed of two segments of linear regressions that intersects at the breaking point (Table 2). For describing a SAR curve with a pattern of increasing first and decreasing latter (i.e., as described by PLEC model), it is expected that the first linear-regression segment has a positive slope k_1 while the second linear-regression segment has a smaller positive slope k_2, i.e., $k_1 > k_2 > 0$. However, this is not the only case for creating PLEC-like changing trend. If the first segment of linear regression before the breaking point of the BR model shows a negative slope ($k_1<0$), it is also possible to obtain increasing-first-decreasing-latter curve shape (after taking the exponential of the log-transformed linear models) when the second linear-regression segment had a smaller negative slope. That is, $k_2 < k_1 < 0$. In both cases, regardless of the slope sign, $k_2 < k_1$. This is the criterion of obtaining MaxAD using BR model (Figures S2A-S2B).

The criterion of obtaining MinAD using BR model can be similarly derived (Figures

S2C-S2D).. That is, for showing decreasing-first while increasing-latter curvilinear trend (as described by PLIEC model), it is expected that the first linear-regression segment has a negative slope k_1 while the second linear-regression segment has a positive slope k_2, i.e., $k_1 <0< k_2$. However, this is not the only case of obtaining PLIEC-like changing pattern. If the first segment of linear regression before the breaking point of the BR model shows a positive slope ($k_1>0$), it is also possible to obtain decreasing-first-increasing-latter curve shape (after taking the exponential of the log-transformed linear models) when the second linear-regression segment had a larger positive slope. That is, $0< k_1 <k_2$. In both cases, regardless of the slope sign, $k_1 <k_2$. This is the criterion of obtaining MinAD using BR model.

Table S1. Overview of the habitat preference of the sampled ten host amphibian species in rural areas of Chengdu City of SW China.

**Species**	**Habitat preference**
Amolops mantzorum	aquatic
Fejervarya multistriata	terrestrial
Hylarana guentheri	aquatic
Bufo gargarizans	terrestrial
Rana chensinensis	terrestrial
Polypedates megacephalus	arboreal
Pelophylax nigromaculatus	aquatic
Odorrana schmackeri	aquatic
Quasipaa spinosa	aquatic
Microhyla fissipes	aquatic

Figure S1. Comparison of inflection point versus extreme value point (MinAD) in the log-PLIEC SAR model by varying the parameter z while fixing the other parameters as c=1 and d=0.05.

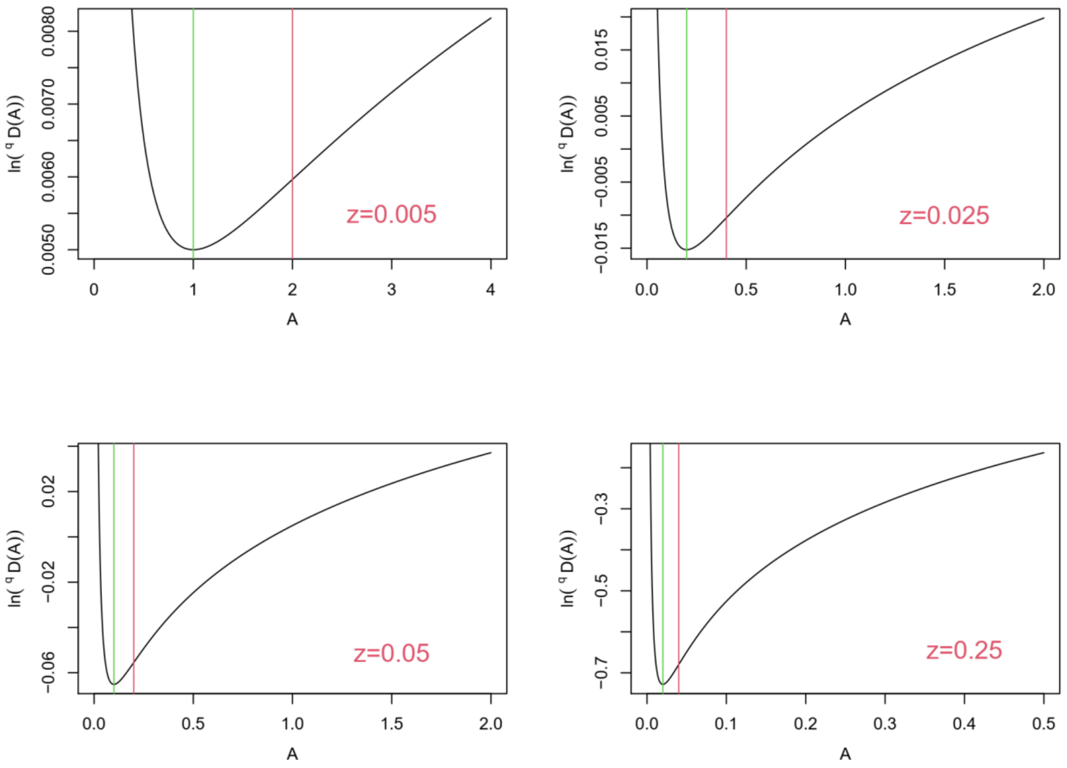

Figure S2. Determination on the existence of MinAD or MaxAD using BR model. In subplot A-B, MinAD occurs (at the origin), while in the subplot C-D, MaxAD occurs (at the origin). The BR model is indicated the solid line while the dashed line is used for comparing the slopes of the two regression line segments.

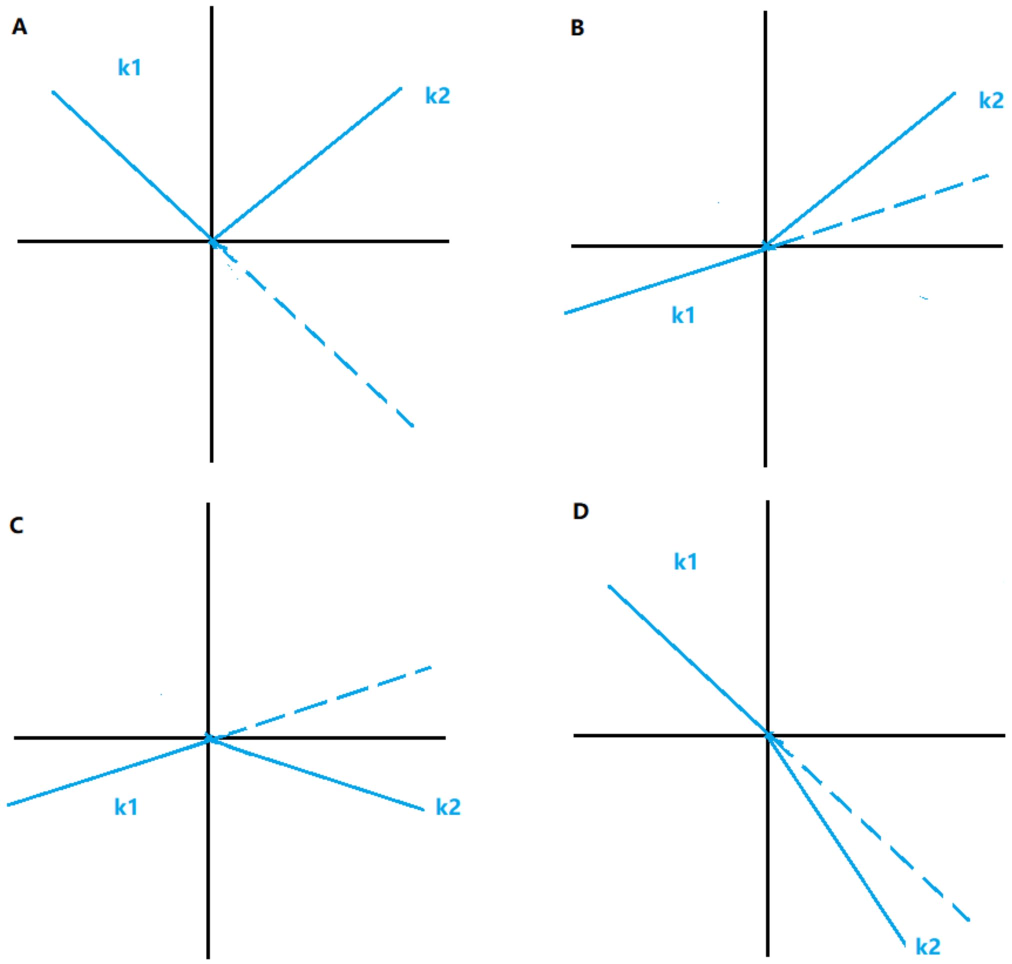

Figure S3. Best fitted SAR models for skin microbes from specimens of host species *Pelophylax nigromaculatus*. Here, skin area size is measured using both 2D and 3D calculation methods and microbial diversity is measured using Hill number with varying orders. For each equally best model, the numeric value after the model name indicates the R^2 value.

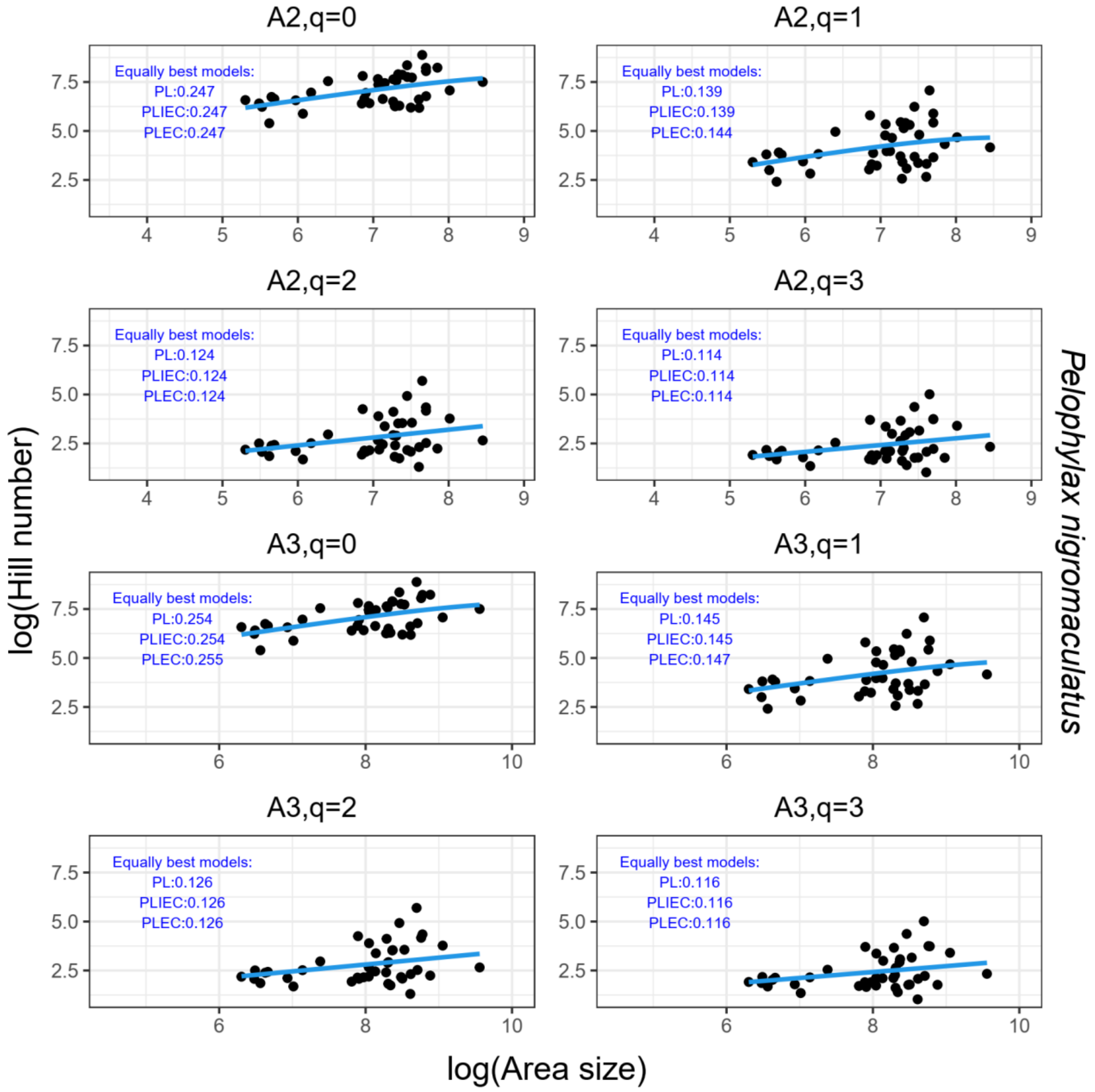

Figure S4. Best fitted SAR models for skin microbes from specimens of host species *Fejervarya multistriata*. Here, skin area size is measured using both 2D and 3D calculation methods and microbial diversity is measured using Hill number with varying orders. For each equally best model, the numeric value after the model name indicates the R^2 value.

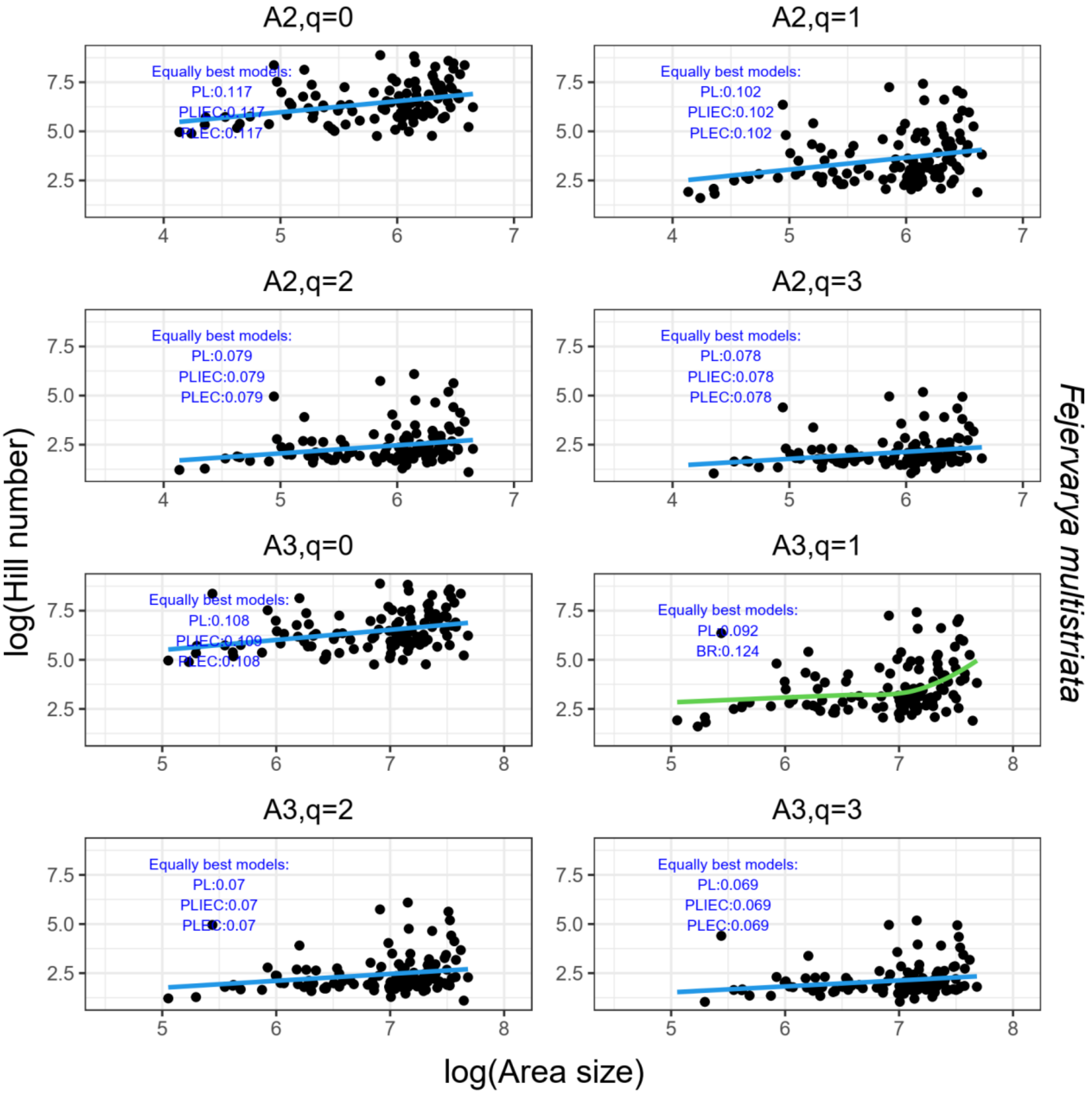

Figure S5. Best fitted SAR models for skin microbes from specimens of host species *Odorrana schmackeri*. Here, skin area size is measured using both 2D and 3D calculation methods and microbial diversity is measured using Hill number with varying orders. For each equally best model, the numeric value after the model name indicates the R^2 value.

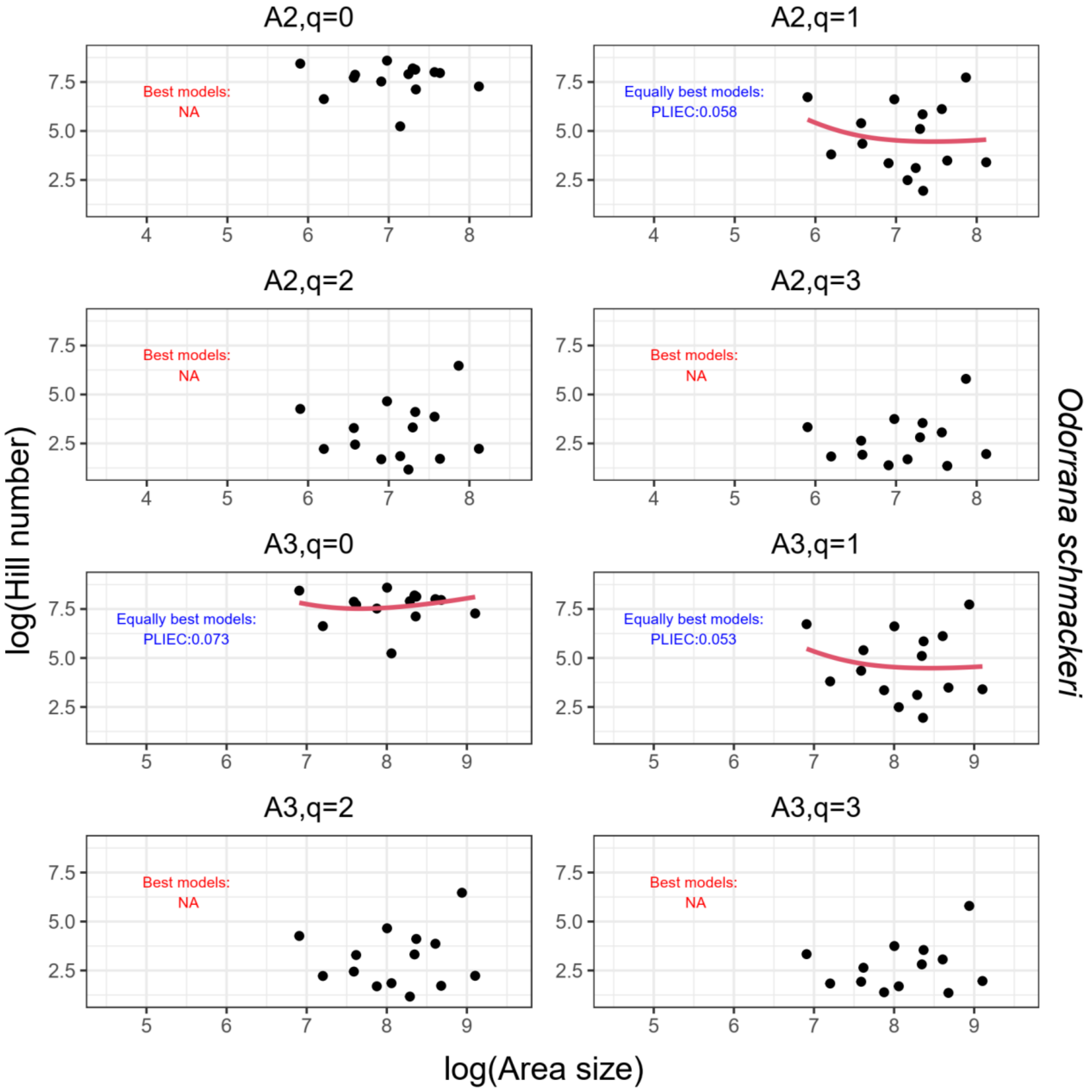

Figure S6. Best fitted SAR models for skin microbes from specimens of host species *Bufo gargarizans*. Here, skin area size is measured using both 2D and 3D calculation methods and microbial diversity is measured using Hill number with varying orders. For each equally best model, the numeric value after the model name indicates the R^2 value.

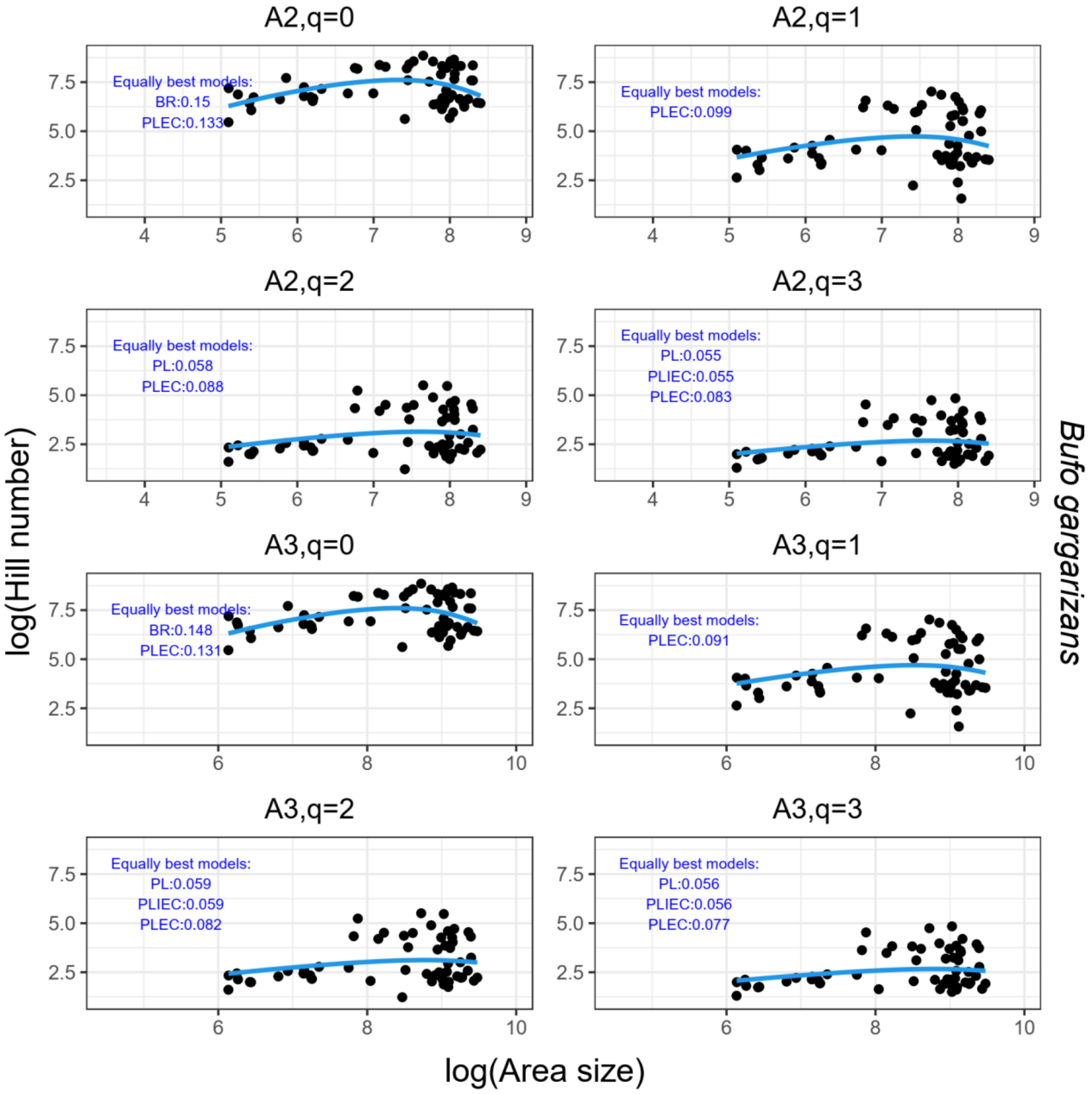

Figure S7. Best fitted SAR models for skin microbes from specimens of host species *Polypedates megacephalus*. Here, skin area size is measured using both 2D and 3D calculation methods and microbial diversity is measured using Hill number with varying orders. For each equally best model, the numeric value after the model name indicates the R^2 value.

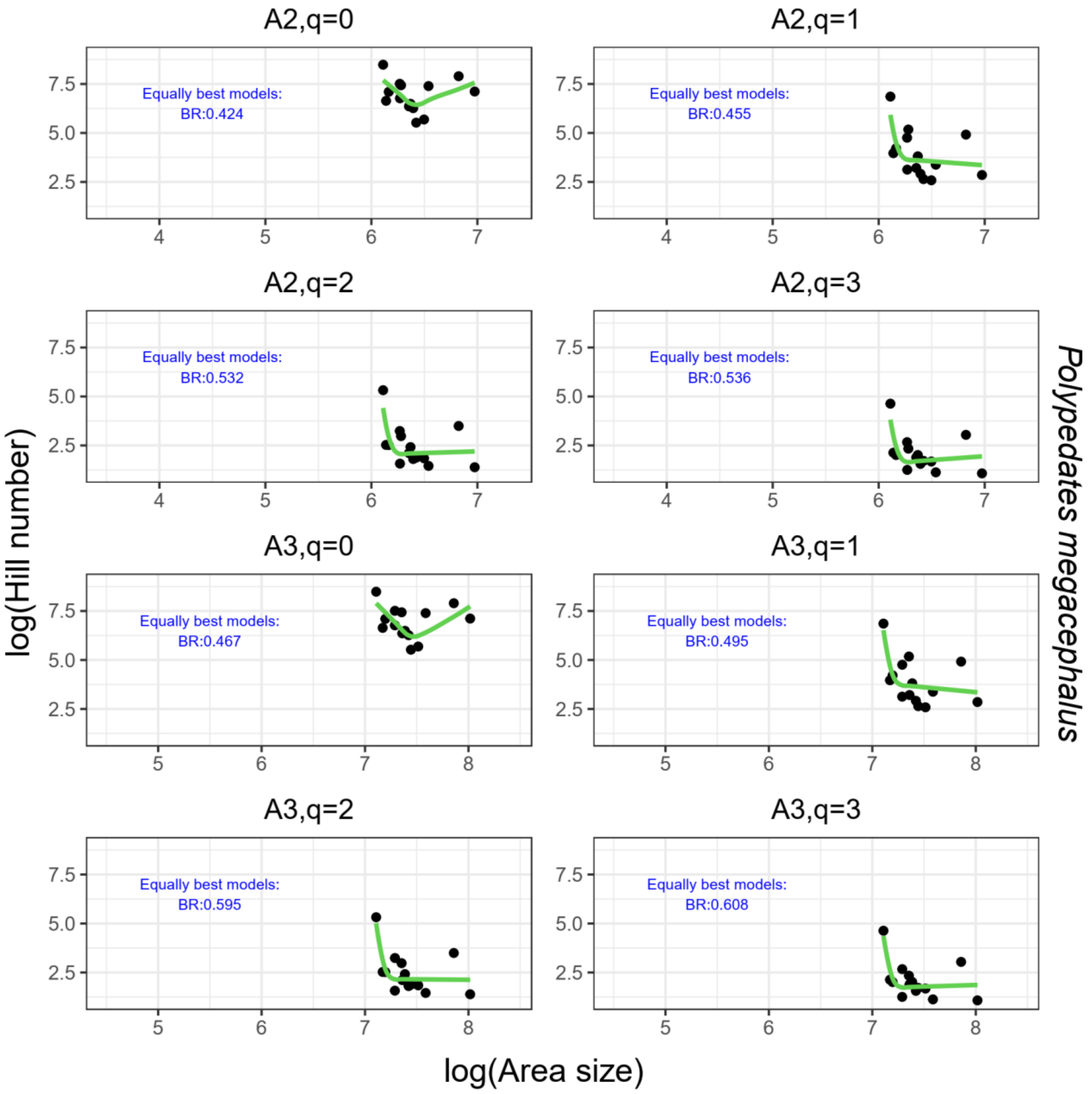

Figure S8. Best fitted SAR models for skin microbes from specimens of host species *Hylarana guentheri*. Here, skin area size is measured using both 2D and 3D calculation methods and microbial diversity is measured using Hill number with varying orders. For each equally best model, the numeric value after the model name indicates the R^2 value.

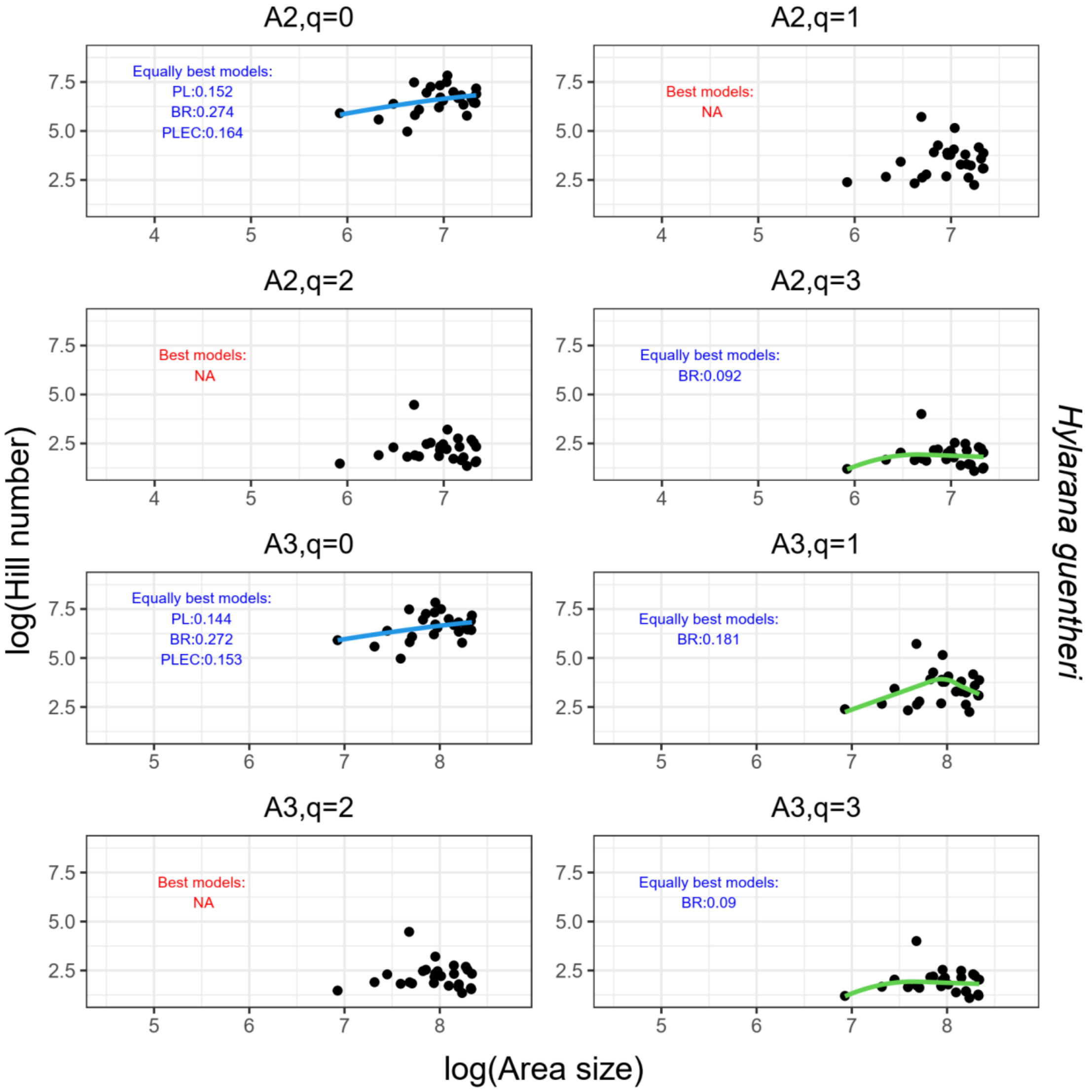

Figure S9. Best fitted SAR models for skin microbes from specimens of host species *Quasipaa spinosa*. Here, skin area size is measured using both 2D and 3D calculation methods and microbial diversity is measured using Hill number with varying orders. For each equally best model, the numeric value after the model name indicates the R^2 value.

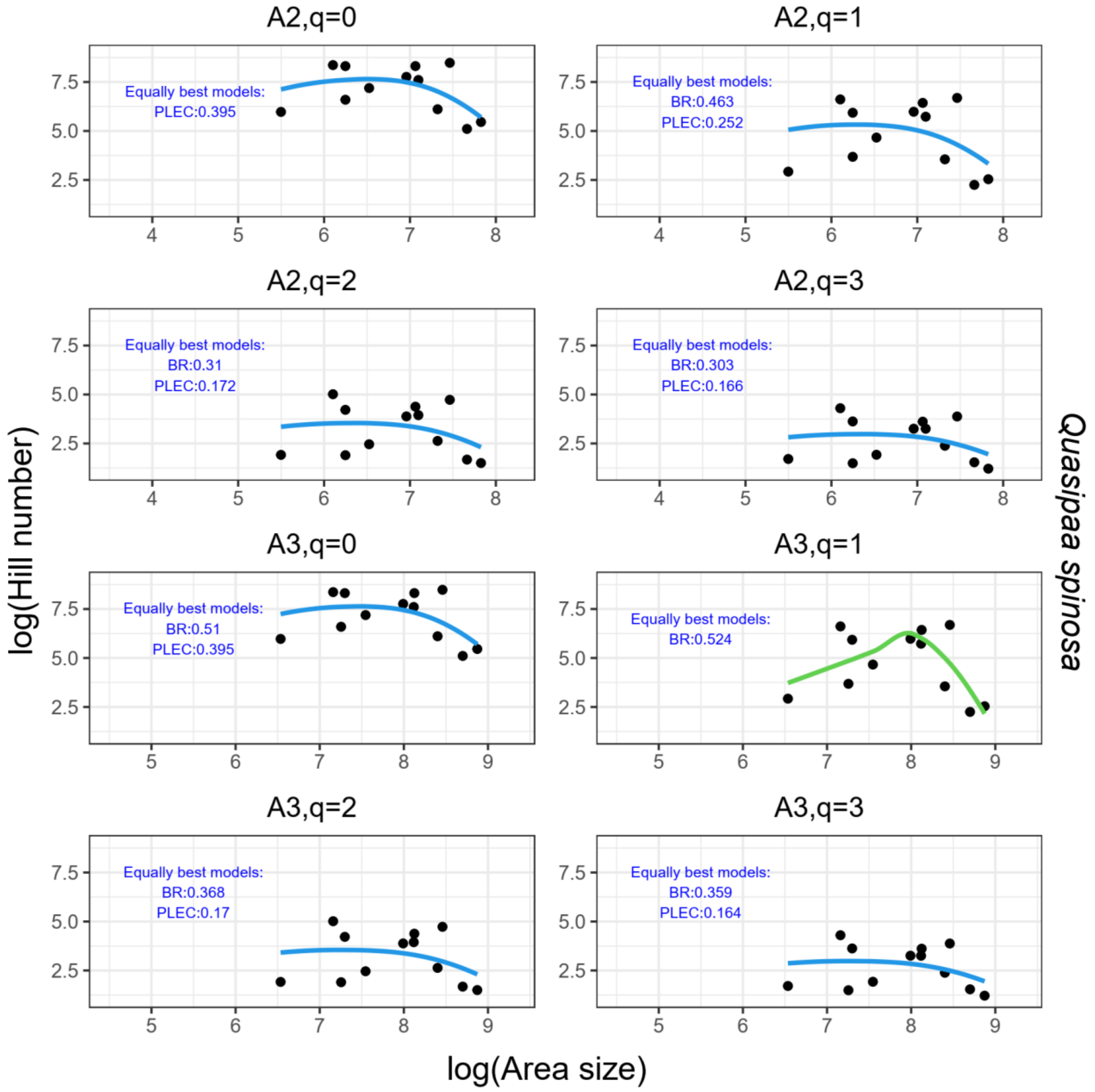

Figure S10. Best fitted SAR models for skin microbes from specimens of host species *Microhyla fissipes*. Here, skin area size is measured using both 2D and 3D calculation methods and microbial diversity is measured using Hill number with varying orders. For each equally best model, the numeric value after the model name indicates the R^2 value.

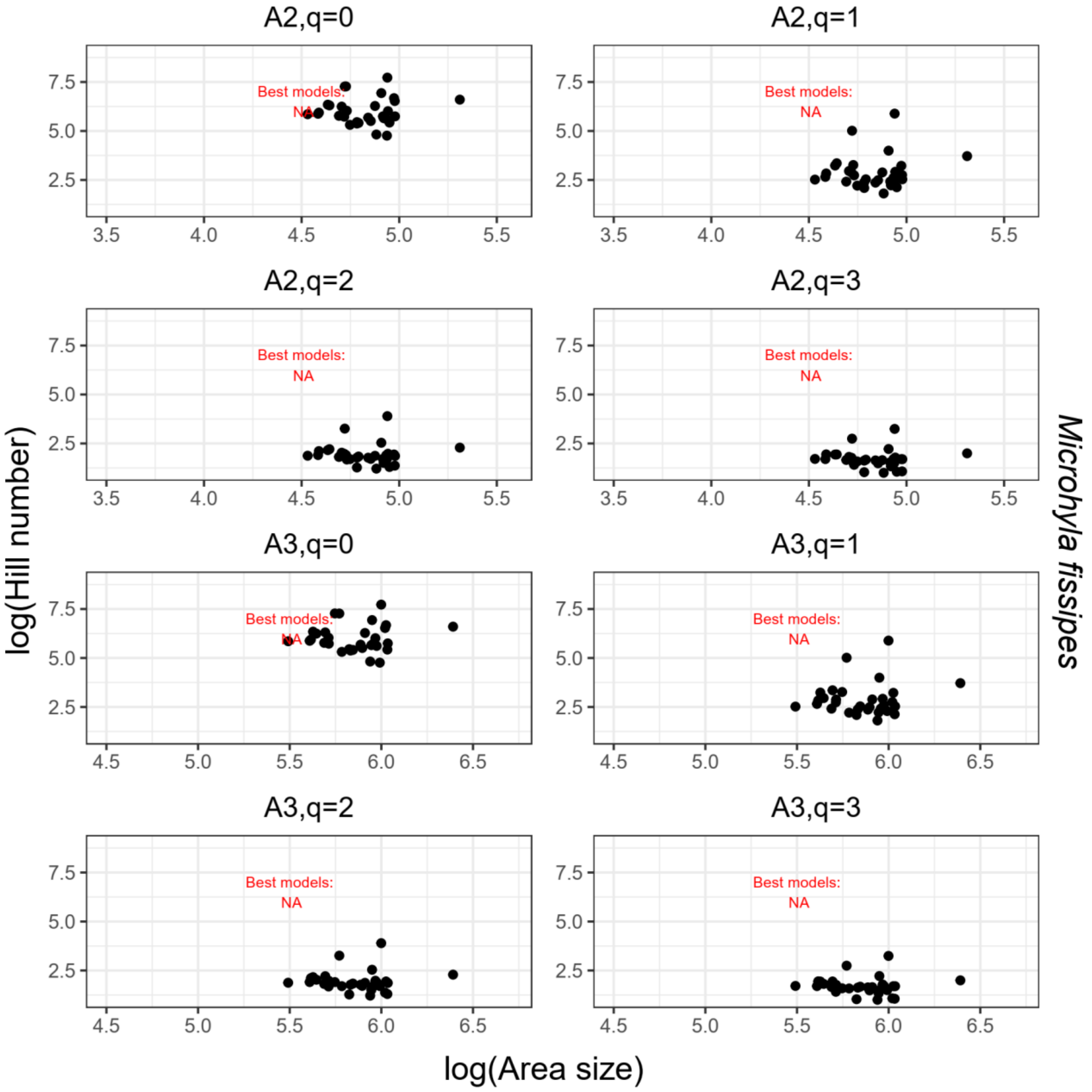

Figure S11. Best fitted SAR models for skin microbes from specimens of host species *Rana chensinensis*. Here, skin area size is measured using both 2D and 3D calculation methods and microbial diversity is measured using Hill number with varying orders. For each equally best model, the numeric value after the model name indicates the R^2 value.

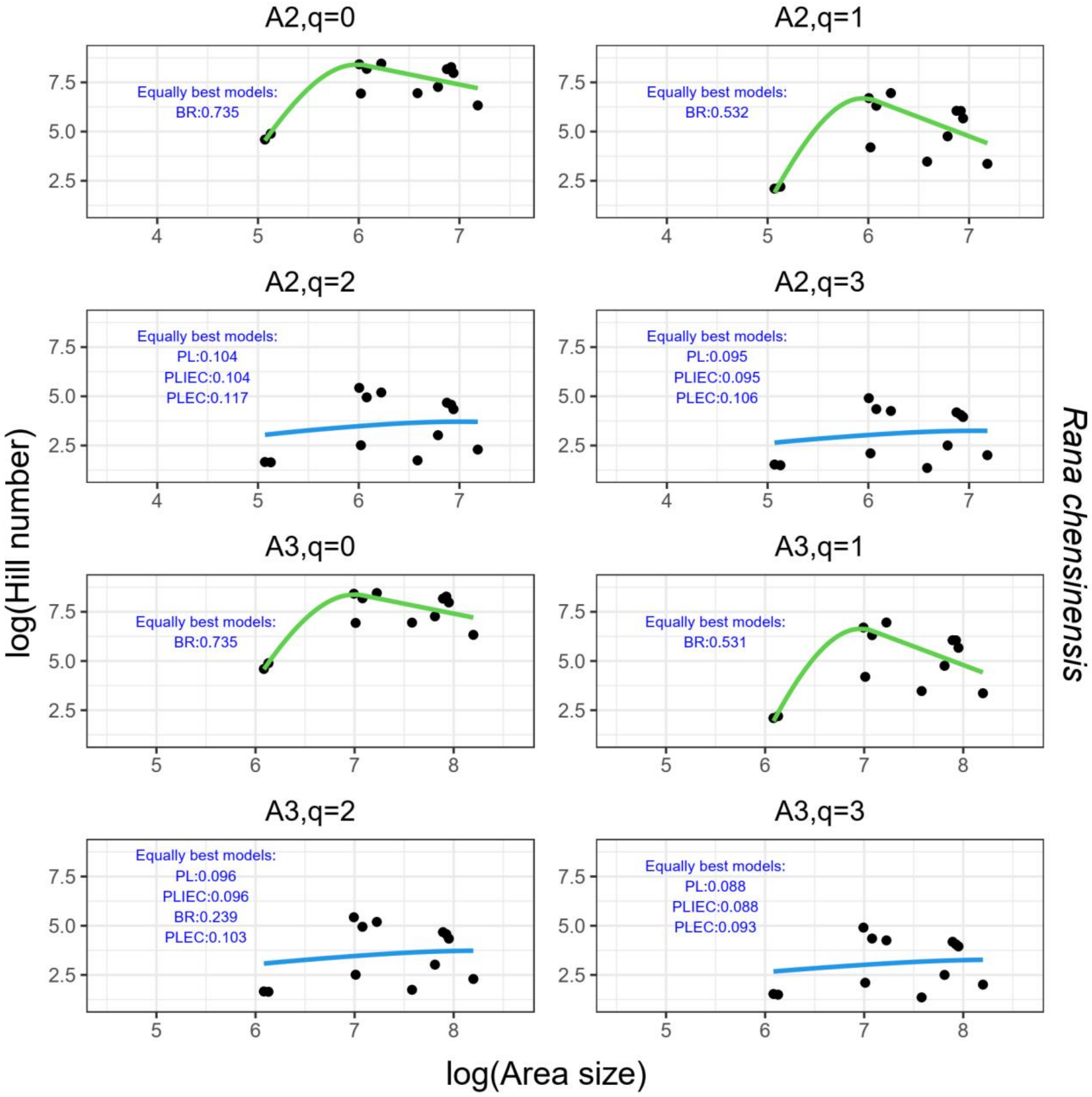

Figure S12. Best fitted SAR models for skin microbes from specimens of host species *Amolops mantzorum*. Here, skin area size is measured using both 2D and 3D calculation methods and microbial diversity is measured using Hill number with varying orders. For each equally best model, the numeric value after the model name indicates the R^2 value.

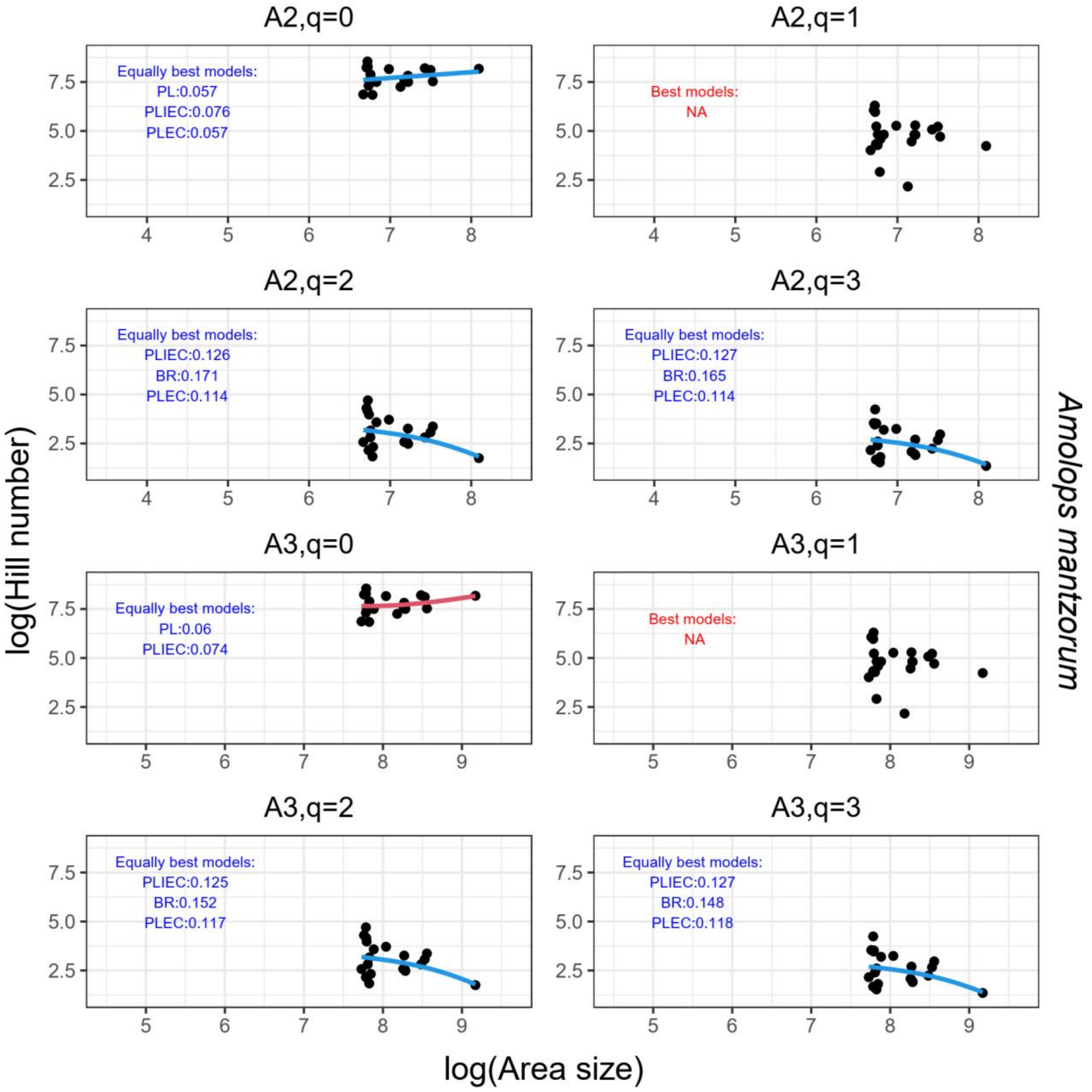

Supplement: Supplemental file 1 — Supplemental material. Download spectrum.01771-22-s0001.pdf, PDF file, 2.3 MB [file spectrum.01771-22-s0001.pdf]
